# Deep learning methods in metagenomics: a review

**DOI:** 10.1099/mgen.0.001231

**Published:** 2024-04-17

**Authors:** Gaspar Roy, Edi Prifti, Eugeni Belda, Jean-Daniel Zucker

**Affiliations:** 1 IRD, Sorbonne University, UMMISCO, 32 avenue Henry Varagnat, Bondy Cedex, France; 2 Sorbonne University, INSERM, Nutriomics, 91 bvd de l’hopital, 75013 Paris, France

**Keywords:** microbiome, metagenomics, deep learning, neural network, embedding, binning, disease prediction

## Abstract

The ever-decreasing cost of sequencing and the growing potential applications of metagenomics have led to an unprecedented surge in data generation. One of the most prevalent applications of metagenomics is the study of microbial environments, such as the human gut. The gut microbiome plays a crucial role in human health, providing vital information for patient diagnosis and prognosis. However, analysing metagenomic data remains challenging due to several factors, including reference catalogues, sparsity and compositionality. Deep learning (DL) enables novel and promising approaches that complement state-of-the-art microbiome pipelines. DL-based methods can address almost all aspects of microbiome analysis, including novel pathogen detection, sequence classification, patient stratification and disease prediction. Beyond generating predictive models, a key aspect of these methods is also their interpretability. This article reviews DL approaches in metagenomics, including convolutional networks, autoencoders and attention-based models. These methods aggregate contextualized data and pave the way for improved patient care and a better understanding of the microbiome’s key role in our health.

## Data Summary

All supporting data, code and protocols have been provided within the article, through supplementary data files or on the following link : https://github.com/CorvusVaine/analyzing_connected_papers_articles.git. This repository contains code used for selection and analysis of articles as well as files generated through these analyses.

**Impact Statement**

Microbiomes are very diverse and complex ecosystems, and the comprehension of their vast diversity is key for both health and environmental studies. In our study, we look at the vast world of research in metagenomics, the study of genetic material from environmental or host samples, spurred by the increasing affordability of sequencing technologies. Navigating through the vast amounts of data generated is not an easy task. Traditional methods hit roadblocks due to the unique nature of metagenomic data. That is where deep learning (DL), a today well-known branch of artificial intelligence, comes in. DL-based techniques complement existing methods and open new avenues in microbiome research. They are capable of tackling a wide range of tasks, from identifying unknown pathogens to predicting disease based on a patient’s unique microbiome and will therefore play a central role in metagenomic analysis. In our article, we provide a comprehensive review of different DL strategies for metagenomics, including convolutional networks, autoencoders and attention-based models. Our goal was to give a precise and comparative overview of the ecosystem of these tools: their goals, data, methods and results. We are convinced that these techniques significantly enhance the field of metagenomic analysis in its entirety, paving the way for more accurate data analysis and, ultimately, better patient care.

## Introduction

A large number of micro-organism species (bacteria, viruses, archaea, fungi and protists) dwell in very different environments. This entire habitat, including the micro-organisms, their genomes and the surrounding environment, is referred to as the ‘microbiome’, while the entire genetic material is referred to as the ‘metagenome’ [1].

Microbiomes can be observed in very different contexts, ranging from environmental microbiomes, such as those found in soils or water [2,3], to communities of microbes living inside the bodies of other living beings [4]. These very dissimilar environments imply significant and varied differences between communities, and therefore specific problems. Host-associated microbiomes tend to be less diverse [5], and their composition evolves in symbiosis with their host, interacting with it and performing functions related to the body in which they evolve [6]. Their metagenomic analysis also results in substantial amounts of host-related data, requiring a specific step of bioinformatic work to distinguish between host data and microbiome data that is particularly critical in low microbial biomass environments, leading to conflicting results between studies [7,8], as well as privacy concerns related to the host genome when studying these ecosystems [9]. The gut microbiome, for instance, plays a key role in the functioning of our own organism and is considered a ‘super-integrator’ of patient health [10]. Lack of microbial diversity is an indicator of chronic host disease [11,13], but also of the health evolution after an intervention [14,15]. It is therefore important to develop tools that allow us to characterize and understand both its composition and its links with human health and disease.

It should be noted, however, that despite these differences between environmental and host microbiomes, the associated data share the same structure, and the analysis methods we describe can therefore be applied to each other with little adaptation. The methods we have reviewed focus primarily on host microbiomes, and we will too, but some environmental microbiome analyses were also encountered and found to be of interest. Therefore, we will be specific when the data analysed are environmental samples.

### Microbiome data acquisition technologies

**Fig. 1.**
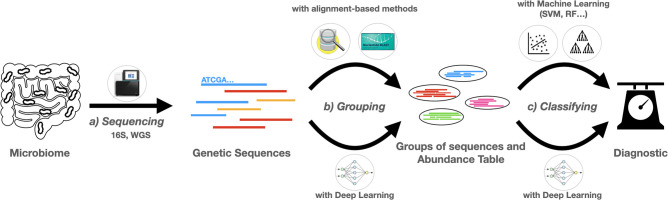
Illustration of the use of deep learning in disease prediction from metagenomic data. The classic simplified pipeline for disease prediction from microbiome data follows three distinct steps. In step (a), high-throughput sequencing of DNA libraries from samples generates millions of reads (from whole genomic DNA in WGS metagenomics or from targeted 16S rRNA genes in targeted metagenomics) from the organisms that make up the community. Second, in step (b), the sequences are either clustered or classified into different groups to characterize the different species present in the sample. This step can be realized by classical bioinformatics pipelines, such as alignment-based methods, or by more recent DL architectures, both of which can be used to estimate their relative abundance. In step (c), the abundance table or the embeddings extracted from the use of neural networks can be used to classify the metagenomes as coming from patients with the disease state or not. DL methods can also be used to integrate additional information (annotations, genes, phylogeny) to classify sequences or metagenome profiles.

Advances in next-generation sequencing (NGS) technologies have boosted the characterization of entire ecosystems and have accelerated the now rapidly growing field of metagenomics. Two main approaches (shown in the first step of Fig. 1) are widely used to characterize microbial communities with high-throughput sequencing: marker-gene metagenomics and whole-genome sequencing (WGS) metagenomics [16].

Marker-gene metagenomics consists of sequencing specific regions of different marker genes (16S rRNA gene for prokaryotes, 18S rRNA gene for eukaryotes or internal transcribed spacer (ITS) for fungi] that allow us to characterize the composition of specific taxonomic groups of the sample. Sequencing a relatively short DNA region requires a low number of reads, resulting in inexpensive analyses. This method has been pivotal in the characterization of microbial ecosystems and is still widely used in quantitative metagenomic studies, despite known drawbacks associated with the variability in diversity estimates and taxonomic resolution of different hypervariable regions [17,18], the lack of resolution at lower taxonomic levels than genus and the fact that functional information about the ecosystem can only be indirectly inferred [19]. Efforts in sequencing full-length marker genes with third-generation sequencing technologies show better taxonomic resolution [20]. Moreover, other lineages such as viruses cannot be profiled by such targeted metagenomic approaches due to the lack of universal marker genes.

Whole genomics sequencing (WGS) metagenomics sequences and analyses the entire genomic content of all organisms in the sample. This makes it possible to characterize the full diversity of the ecosystem, including archaea, bacteria, viruses and eukaryotes. WGS data are highly resolving and more complex, allowing differentiation down to the strain level as well as direct functional potential profiling [21,22].

There are different sequencing platforms that produce various types of reads, mostly varying in length and precision [23]. Platforms such as Illumina [24] produce mostly short reads (150–300 bp), needing fragmentation and amplification for large strand analysis. PacBio [25] or Nanopore [26] produce long reads (with an average length between 10 and 30 kb and up to millions of sequences). However, Nanopore sequencing in particular tends to be more error prone, even if recent breakthroughs with updated flowcells and chemistries have substantially improved the sequence quality, making them more reliable than before [27,28]. Nevertheless, most metagenomics data generated today are still from WGS-based short reads.

All these sequences are then analysed to achieve different goals. A first goal may be to identify sequences of interest such as those associated with specific functions. This task will be referred to as ‘functional annotation’. This may involve processing each read individually to search for specific sequences associated with pathogens or other global functions.

However, a primary challenge in metagenomics is also to identify which micro-organisms are actually present in the sample. This can be achieved by either performing *de novo* metagenomic assembly of metagenomic reads or assembly-free approaches where metagenomic reads are used directly for taxonomic and functional profiling based on reference databases.

In *de novo* metagenomic assembly, raw reads are first assembled into longer and more contiguous sequences (contigs) that can be subsequently grouped, or ‘binned’, either in a supervised manner using alignment to reference sequences [29], or in an unsupervised manner, independent of reference sequences, exploiting other sources of information like compositional profiles such as k-mer distribution and abundance profiles [30,33]. By binning contigs, it is possible to reconstruct a metagenome-assembled genome (MAG), which represents whole or part of the genomes of species present in the sample. [33]. In this context, the human gut microbiome is one of the microbial ecosystems that has been more extensively characterized at the genomic level, with several large-scale metagenomic assembly studies yielding comprehensive catalogues of human gut MAGs [34,36]. When using MAGs, it is also possible to calculate the relative abundance of each MAG in the metagenome by considering the number of reads mapped to an MAG. In both cases, this results in an abundance table representing the metagenome based on the abundance of each species. Another approach is to start by building representative, non-redundant gene catalogues [37,38], which are themselves binned to metagenomic species (MGS) [33,39]. At the end of this step, the output is an abundance table linking each taxon to its metagenomic gene abundance (MGA).

Other methods, called ‘assembly-free methods’, start by grouping together the reads that belong to a particular taxonomic unit, such as species. They exploit sequence similarity [29,40, 41] or k-mer content similarity [30,42] against reference databases. For example, reads are aligned against gene markers for taxonomic profiling [43] or comprehensive gene catalogues that maximize the genomic knowledge of microbial ecosystems, such as Genome Taxonomy Database (GTDB) [44], the Global Microbial Gene Catalog (GMGC) [45] or the Kyoto Encyclopedia of Genes and Genomes (KEGG) [46]. This provides a representation of the composition of a metagenome as well as its functional potential.

Traditional bioinformatics methods have several drawbacks: they are computationally expensive, affected by sequencing errors and often dependent on reference databases. However, the majority of the micro-organisms found in the human microbiome remain poorly characterized. To this day, machine learning (ML) methods such as SVM or random forest-based methods have proven their efficiency and are alternatives to alignment-based methods to classify sequences [47]. Although they cannot correct sequencing errors, the ever-increasing size of the models might be able to capture the different possible variations in DNA, if trained with enough data (in terms of quantity, but also quality and diversity). Moreover, even if deep learning (DL) models can be heavy and long to train, inference can be very fast once training is over, which could be an interesting advance in bioinformatics.

Handling reads to obtain a quantification of the microbiome can be referred to as ‘quantitative methods’. Once the abundance table of the metagenome is obtained, it can be used for microbiome classification analyses. More specifically, this consists of establishing links between the metagenomic data obtained in the first step and sample information such as disease status or severity in the case of a host-associated microbiome. A brief summary of these steps is illustrated in Fig. 1.

### Deep learning approaches

The task of predicting patient phenotype can be addressed using various ML models. With an increasing number of public example datasets, these algorithms can learn and extract important patterns from the data in order to classify samples based on their various characteristics. DL is a specific branch of ML that focuses on algorithms based on layers of artificial neurons that receive and process information from previous layers of neurons [48]. Data are channelled through the network to generate an output, facilitating the learning process as the network adjusts the neuron weights via backpropagation of errors. The most notable strides empowered by DL are discernible in domains like image recognition and natural language processing (NLP).

DL stands out for its superior performance on large datasets, outdoing many other ML algorithms that reach a performance plateau with a given quantity of data. Furthermore, DL techniques possess a robust capacity to unearth intricate features, often imperceptible to human observation, which is called ‘representation learning’. DL can also perform various learning paradigms (unsupervised [49], semi-supervised [50], multiple instance learning [51]). These paradigms allow different types of learning: exploring the data in a certain direction with supervised learning, or letting the network do the task to draw conclusions with unsupervised learning. In particular, the ability to learn mathematical representations from the data, such as numerical vectors called ‘embeddings’, makes it possible to group or mathematically classify different samples or observations. An embedding is a low-dimensional numerical vector representation of high-dimensional data, capturing semantic and syntactic relationships between the elements being embedded. They are used to translate high-dimension data that would be difficult to work with for an ML model. Embeddings can be used for clustering or classification.

Various types of neural networks (NNs) find extensive application in metagenomics, and we can cite a few of them here.

The conventional feed-forward neural network (FFN), also known as the multi-layer perceptron [52], is unidirectional, with each layer comprising a specific number of neurons interconnected to all neurons in the preceding and succeeding layers. Therefore, information flows in only one direction, from the input nodes to the output nodes and through hidden nodes (if the network contains any). The error is then back-propagated through all the connections between nodes, allowing the network to correct itself. FFNs are considered a simple architecture and are often used as building blocks or parts for other networks. However, this type of network faces difficulties with complex data in terms of overfitting [53] and vanishing gradients [54].

Convolutional neural networks (CNNs) [55] are well known for their performance in image classification. Inspired by the cortex of vertebrates, they use filters based on the operation of convolution that move across data, extracting local spatial features. In the case of metagenomics, they can be used to classify sequences with common local patterns [56], such as common nucleotide patterns, but also to characterize the structure of the microbiome [57].

Recurrent neural networks (RNNs) [58], with the introduction of cycles in connections, are well suited for temporal or sequential data processing. Today, most widely used versions of RNNs are the long short-term memory neural network (LSTM), which perform better at detecting long-term dependencies, or gated recurrent units (GRUs), which can input or forget certain units [59]. For example, these networks can be employed to analyse DNA as sequences, allowing predictions about the presence of specific DNA elements, such as phages [60]. Or they can be used to analyse the abundance of microbial species through time to predict, for instance, the evolution of the microbial ecosystem [61, 62].

Autoencoders are a type of NN designed to distill pertinent features from input data [63]. Their operation involves dimensionality reduction of the input data (encoding) followed by its reconstruction from the encoded data (decoding). The encoded data are then supposed to be a contextual representation of the input data containing features of interest for classification, making it simpler and more suitable for ML algorithms, but also to underline important features in the data that would not be easy to uncover otherwise. There are many types of autoencoders using various processes (variational [64], convolutional [65]). When analysing abundance tables, autoencoders can perform crucial roles in overcoming the challenges of high-dimensional data [66], but they can also represent DNA sequences and features in a manner suitable for metagenome binning [67].

Another field where DL has shown remarkable results is NLP, focused on the interactions between humans and computers using natural language. Researchers have explored ways to represent, understand, analyse and generate language with artificial intelligence (AI). The biggest advances have come with the use of transformers [68], a type of DL model that relies on attention mechanisms to find coherence between different parts of data, one of the most famous applications being to encode the data contained in a sentence through the relations between its elements. In particular, generative models have been very successful today because of their ability to generate data, such as text and images, and not just classify it. These models mostly rely on this architecture.

A primary challenge in DL is the need for substantial volumes of data to train models. Given that these models comprise millions to billions of parameters, they necessitate a large number of examples to autonomously discern abstract features. In addition to procuring costly medical data, several strategies are adopted such as data augmentation or data generation methods. In addition, the high dimensionality of the data relative to the number of samples, its high sparsity and its compositionality can be challenging for ML algorithms. We will discuss these challenges and how they can potentially be mitigated.

A critical challenge in the medical domain is not only establishing a diagnosis, but also understanding the rationale behind it. This understanding aids in contrasting the diagnosis with a practitioner’s personal knowledge and bolsters their confidence in the outcomes. The ‘black box’ characteristic of DL models presents an obstacle here. The complexity of these models obscures the logic driving their decision-making process, underlining the significance of ‘interpretability’ in the field of DL [69]. Some interesting reviews of these methods have already been published [70,71].

In this review, we will present different DL methods used in metagenomics and analyse their motivation, qualities and drawbacks. This study focuses on the task of metagenome classification, and more precisely in the case of host-associated microbiomes, of disease prediction itself, which is closely related to the issues of sequence classification (binning, taxonomy, identification) and ultimately phenotype prediction. Therefore, our work covers all steps and tasks performed for the analysis of the human metagenome in this context. Although various reviews on DL in metagenomics exist [72,75], none have studied all methods from raw reads to disease prediction, and they either include shallow ML and do not focus on DL, or focus on a specific metagenomic task (phenotype prediction, binning or sequence classification).

## Methods

**Fig. 2.**
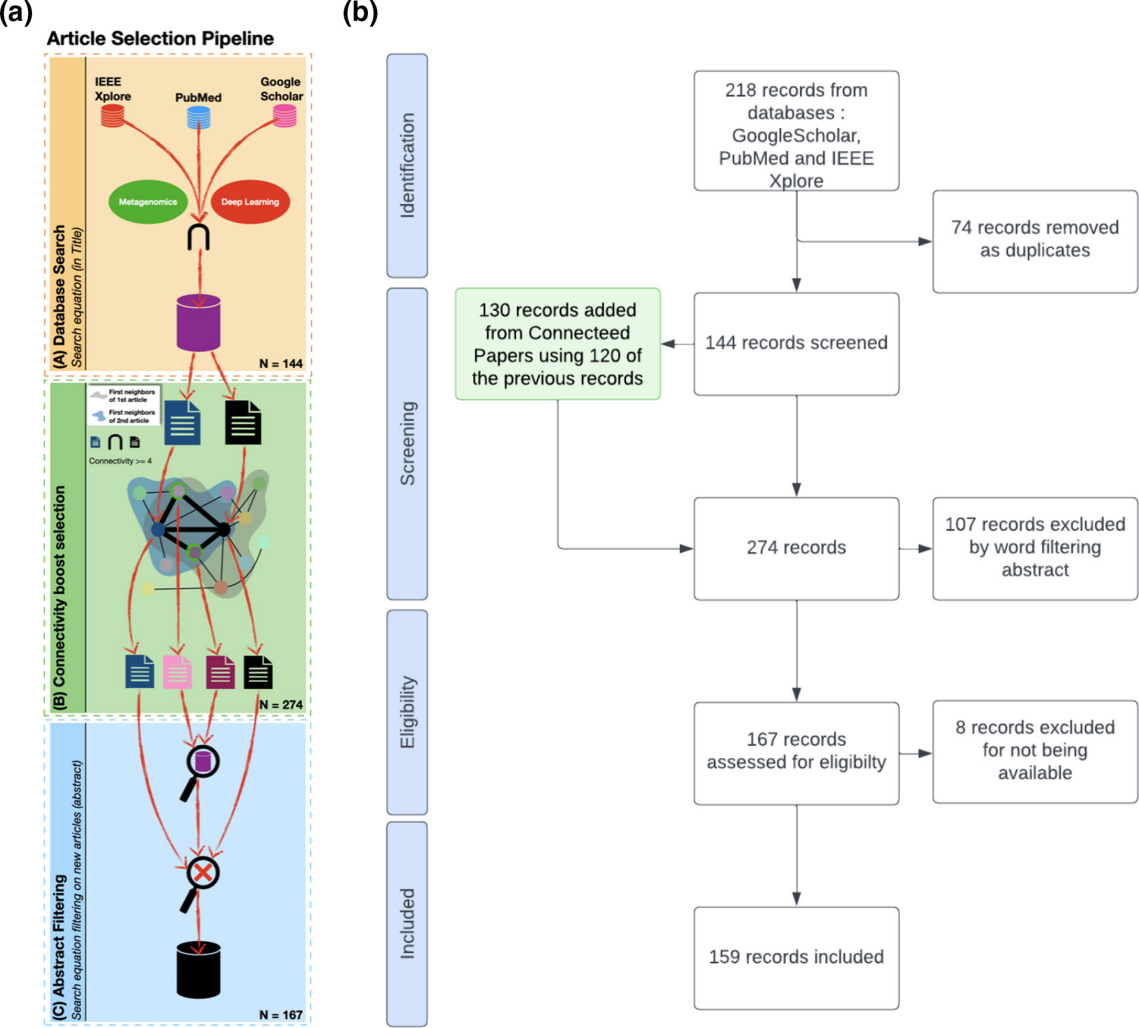
Article selection methodology used in this paper. (a) The pipeline of our methodology for choosing articles. It consists of three steps. (A) Articles are extracted from three databases using our research equation. (B) Remaining articles are provided as anchors to Connected Papers, which generates similarity graphs for each article. Once retrieved, the graphs are integrated in a unified graph. Articles with a certain number (that we will set to 4) of links pointing towards them are added to the selection. (C) The newly added articles are filtered using the same research equation as in step (A), but searching words in keywords and abstract instead of title. Numbers correspond to the second phase of screening. (b) PRISMA-type diagram for article selection of this review. The method developed here enriches the research equation selection with Connected Papers; this diagram represents the selection along with this enrichment in green.

Our selection of articles of interest aimed to follow strict and reproducible rules to analyse these very active fields. The pipeline of our review selection is described in Fig. 2(a).

### Review search equation

**Fig. 3.**
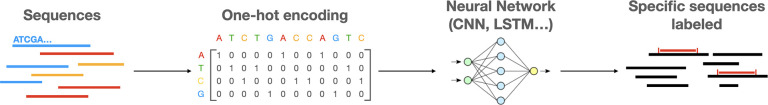
Sequence mining workflow diagram. DNA sequences are encoded, most of the time with one-hot encoding, which leaves a matrix of dimensions 4 by the length of the sequence. The sequence is then analysed by a neural network, often a CNN, to be classified as a specific type of gene, for instance a viral sequence. Adapted from: [83].

The first step (step A in Fig. 2a) consists of searching articles in three different bibliometric databases (Google Scholar, PubMed and IEEE Xplore). This research includes articles until July 2023 based on the following equation that tries to cover both metagenomics and DL concepts in the article tittle, summarized in Fig. 3:

Allintitle: (metagenome OR metagenomics OR metagenomic OR microbiome) AND (‘deep learning’ OR ‘neural network’ OR embedding OR interpretable OR autoencoders OR CNN OR convolutional OR LSTM OR ‘long short-term memory’ OR NLP OR ‘Natural Language Processing’ OR transformer OR BERT)

This allowed us to identify 142 relevant references (Google Scholar), 56 articles (PubMed) and 20 articles (IEEE Xplore). By removing the duplicates, we obtained 144 unique articles after this screening step.

### Automatic enrichment with the Connected Papers tool

Connected Papers software (https://www.connectedpapers.com/) was subsequently used to enrich the initial set of 144 articles by searching for closely related articles using a similarity measure based on co-citation and bibliography over an enriched database of more than 240 million papers (Semantic Scholar; Step B in Fig. 2a).

This process allowed us to fetch up to 2443 new articles that were not captured by the restrictive search described in step 1 of the pipeline, for which an integrated co-citation article directed graph was constructed, where connectivity ranged from 1 to 34. For each Connected Papers graph the raw list of articles was obtained.

Based on this integrated co-citation graph, 130 additional articles with co-citation connectivity >4 were included in our review. This threshold was fixed in order to limit the additional articles and reject as few articles as possible while not adding more articles than the original database size. This list was further filtered by applying the initial search equation over title and abstract in order to keep the ones relevant for the present review, yielding 23 supplementary articles that were added to the initial corpus for further analyses. Overall, a total of 167 articles were used for the review. The PRISMA-style diagram synthesizing the evolution of our database is illustrated in Fig. 2(b). The full methodology, supplementary statistics and figures can be found in Tables S1, S3, S4, S5 and S6 (available in the online version of this article).

We decided to add to our dataset the articles with a co-citation connectivity of 4. We chose this threshold because it allowed us to reject as few articles as possible while not adding more articles than the original database size. This methodology is decsribed in the Supplementary Material.

### Filtering new articles

Among the newly discovered articles, it is important to discriminate those that are relevant to the subject. We thus decided to reuse our search equation as a filter for these articles, but this time by searching for keywords in the abstract and the article keywords instead of the title (see step C in Fig. 2a). After filtering, 23 supplementary articles are kept and added to the initial corpus for further analyses.

Overall, a total of 167 articles were used for the review. The PRISMA-style diagram synthesizing the evolution of our database is illustrated in Fig. 2(b). Supplementary statistics and figures can be found in Tables S1, S3, S4, S5 and S6.

## Results

Metagenome classification and metagenome-based disease prediction can be decomposed in two steps, corresponding to two scales, and therefore DL methods can work at the read/sequence level and at the abundance matrix level. In the two subsections below, we review sequence-based methods, respectively methods concerning functional annotation and profiling of a metagenome directly from the sequenced raw reads or generated contigs. Finally, we review the methods used for phenotype classification.

### Deep-learning methods for functional annotation

Functional annotation of metagenomics data allows us to infer the functional potential of natural ecosystems. Traditional bioinformatics approaches utilize sequence similarity of raw reads or predicted genes in metagenomic assemblies with reference databases to do this functional assignment using state-of-the art tools like blast [29], DIAMOND [41], HMMER [76] or more recently MMSeqs2 [77,78]. DL methods have been shown to be extremely powerful in learning patterns from specific functional classes in reference databases using these as prior knowledge to train the network to discover sequences. We can cite DeepARG [79] or Meta-MFDL [80], which classify respectively whether a given sequence is an antibiotic resistance gene or a gene fragment. These models do this by using characteristic genes and ORF features such as ORF coverage, amino acid or codon frequencies, and Z-curve, and form a vector that is then fed into a deep stacking network. In the same way, the ONN method [81] uses extensive information from ontologies to build an ontology-aware NN for gene discovery.

#### Research from raw reads classification

Other DL methods aim to classify whether sequences play a specific role. However, here most of the feature extraction process is performed using the NN rather than relying on prior knowledge. These models encode sequences so that an NN can easily process them. One of the commonly used techniques is one-hot encoding of a sequence. These encoded sequences are then analysed by an NN, which ultimately classifies them. An example is shown in Fig. 3.

This is the case of CNN-MGP [56], which uses a CNN to extract patterns from a one-hot representation of an ORF and classify it as a gene or not, and differentiates between host and microbial sequences. Several methods search for plasmids and phage sequences among metagenomic sequences: tools such as PlasGUN [82], PPR-Meta [83] and DeephageTP [60] claim to achieve better performance than alignment-based methods in detecting phages and plasmids using DNA sequences and/or proteins and analysing them with CNNs. The last in particular outperforms VirFinder [84], a virus identification method that has now been adapted to a DL architecture. In fact, DeepVirFinder [85] was developed using a similar approach. RNN-VirSeeker [86] relies on encoding sequences but considers a sequence as a temporal series and therefore uses a recurrent NN [58]. Although trained on long reads, it performs better on short reads than previous methods because it captures the sequential nature of DNA rather than local features, changing the analysis paradigm. To date, CNNs show the best performance in this type of sequence classification problem.

Some tools, also designed to identify viral sequences, now use more than simple sequence encoding, counting on deeper features. These methods, represented by CHEER [87] and CoCoNet [88], rely on k-mer embedding and computed features (here, k-mer distribution and coverage), respectively. These features, which we will specify and develop later, allow them to achieve state-of-the-art or even better results in viral sequence classification. This is the reason why they are widely used.

#### NLP-based analysis

In the last few years, a new paradigm has emerged in the analysis of metagenomic sequences, very different from those previously covered. They are based on the recent breakthroughs in NLP using attention, word embeddings and transformers, and are applied to DNA. These methods are used to model the meaning of a text by representing various units of a sentence as mathematical vectors. DNA also has its own alphabet with nucleotides, sentences with sequences and even possibly words with k-mers. This analogy opens the way to analysing DNA by adapting NLP methods.

Various methods use sequence embedding techniques to embed their sequences. MetaMLP [89], for example, embeds k-mers with a small alphabet and partial matching, allowing for rapid functional profiling. DETIRE [90] uses methods close to those seen before, but by combining one-hot encoding with TF-IDF embedding of k-mers for virus detection. The structure of the data is also captured with a graph that links k-mers to their original sequences and their label (viral or not). Finally, CNN and LSTM layers aim to capture both spatial and temporal features. Virsearcher [91] also uses word embedding and CNN to analyse the sequence and combines the output with hit ratio of the virus.

Although these methods use word embedding techniques, new DL methods exist using the mechanism of attention.

Attention-based tools and in particular transformers are quite recent, but their application seems well suited for sequence classification. VirNet [92] uses a deep attention model to perform viral identification and claims to achieve state-of-the-art accuracy. Famous transformer models have also been adapted here: ViBE [93] uses a hierarchical BERT model to classify viruses at order level by pre-training it with reference virus genomes. It outperformed alignment-based methods when evaluated on reads simulated from genomes in the same taxa as the genomes from which the simulated reads used for training were generated. Small BERT models were also adapted for identification of antimicrobial peptides [94]. Finally, DLMeta [95] combines both CNN and a transformer to capture both local and global features from sequences, allowing various metagenome identification tasks such as viral identification, but also gene prediction or protein domain prediction.

### Sequence grouping: from reads to metagenome profiling

Here, rather than identifying the type or function of a specific sequence, we focus on methods that allow the user to group sequences/reads into bins and subsequently profile a metagenome (see Introduction). Many non-DL-based methods have been developed to perform such tasks and show impressive results. Many of them allow us to bin contigs into genomes and thus provide a list of species representing the microbiome. We can cite MetaBAT [96] and MetaBAT 2 [16], which use probabilistic distances and tetranucleotide frequencies, as MaxBin [97] and MaxBin 2 [67] do. Finally, a method like GraphBin [98,99] uses assembly graphs and de Bruijn graphs to cluster contigs. On the other hand, some methods use ML to compute taxonomic classification of metagenomic sequences [100]. All of these methods provide good results when binning natural and synthetic datasets, such as CAMI datasets [101]. However, DL methods bring numerous novelties notably in terms of discovering new relevant features and embedded representations.

#### Composition-based methods

The one-hot encoding of a sequence is a limited method with respect to the goal of grouping it with others (binning). Various methods perform binning using autoencoders but relying on one-hot encoding [102,103] or reference database annotations only [104]. However, these methods are now outperformed by methods that provide better sequence representations. Methods working with computed features process a sequence by modifying its representation with features inferred from the reads. K-mer frequency distributions are well known for their utility in characterizing sequences, acting like ‘signatures’. We will refer to these methods as ‘composition-based methods’. The best results are obtained using 4-mers, which corresponds to tetranucleotide frequency (TNF) [105]. (In the case of 4-mers, as reverse-complements are considered as one, the representative vector is of length 136.)

##### Learning representation of reads

Computing an abundance matrix by grouping reads taxonomically is a difficult task as reads are often quite short (100–150 bp). Two paradigms can be distinguished to perform this quantitative analysis: the first one relies on classification methods and the second one on clustering. Classification methods are supervised methods: they process reads, extract their features and output a single class label per read based on those of the training labels (e.g. a taxonomic group) classifying directly at a given level [100,106], or by using a hierarchical classifier to distinguish, for example, first at the kingdom level, then using this result to progressively classify at lower taxonomic levels [107]. The sequence features are treated through various layers, ending with a classification layer (e.g. a SoftMax). Due to the variety of data, there is often a possibility of rejection of a read that is too difficult to analyse. Once the classification is done, the loss is computed and back-propagated through the layers cited above.

**Fig. 4.**
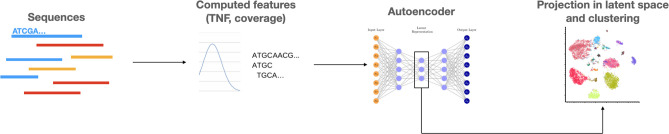
Example of an unsupervised binning method using autoencoder. Features like TNF (tetranucleotide frequency) or coverage are extracted from sequences and analysed by an autoencoder, to create an embedding vector representing the sequence. This vector is then projected in a latent space, allowing visualization and clustering of sequences. Adapted from [118].

The second approach relies on clustering: it is an unsupervised method, very different from classification. Here, the features of the sequences are processed to formulate an embedding vector (Fig. 4). This vector is then projected into a latent space, thereby producing a novel data visualization. These points can be grouped through clustering algorithms such as k-medoids or k-means based on their proximity in the embedding space [104,108]. These groups and their population will form the abundance table. Thus, this method does not rely on pre-selected classes in the program, but on clustering algorithms (which may be seeded with reference sequences). Although this method is not a classification method from the ML point of view, in the context of metagenomics, the two approaches have the same purpose: to group sequences based on their origin and to compute an abundance table.

Different DL architectures can be used to embed the computed features into a vector. To extract features, methods like CNN can be used for taxonomic classification[106,109, 110]. Autoencoders are used by MetaDEC [111], which groups reads together by creating a graph where the nodes are reads, linked if they exhibit significant overlap in their substrings. Subsequently, clusters are extracted from this graph. It then selects a subset of representative reads for each cluster of non-overlapping reads. The k-mer frequency of each subgroup is then used to build representations using autoencoders. These clusters derived from embeddings produced by DL methods outperform the clustering based on principal axis derived from dimensionality reduction techniques, such as principal component analysis (PCA), t-distributed stochastic neighbor Eebedding (t-SNE) or uniform manifold approximation and projection for dimension reduction (UMAP) [112]. They are also very useful because they allow visualization of the data partitioning and are therefore much more interpretable.

##### Metagenome assembly and contig binning

In the context of contig binning, deep learning methods like VAMB [67] have shown proof of concept that DL could outperform state-of-the-art binners like MetaBAT2 [16] or MaxBin2 [113] in classifying contigs from different types of microbiomes from simulated CAMI2 datasets or discovering new closely related strains. VAMB works with contigs and takes as input both the k-mer frequency and the abundance of reads mapped to the contig. These inputs are treated by a variational autoencoder, creating a new feature vector and then mapped to a latent space. This space is then clustered using an iterative medoid algorithm.

Based on the VAMB architecture, various methods have been developed for its extension or the use of other sources of information. First, the authors of CLMB [114] took into account the noise, rarely considered in metagenomic analysis. To do so, they simulated different types of noise, augmenting contig data with noised sequences. The model was trained with the double objective to minimize the reconstruction error between noised versions of a same contig while identifying differences between different contigs. This approach was based on the principles of contrastive learning [115]. Compatible with other binners, CLMB was more refined and outperformed them (MaxBin2, VAMB and MetaBAT2) on the same CAMI2 datasets. AAMB [116], an extension of VAMB, is also based on its architecture and compatible with it. Instead of variational autoencoders, it relies on adversarial autoencoders. The strategy is to use the same input as VAMB and to encode it in two latent spaces: one is continuous and the other categorical. These two spaces are clustered, and a discriminator for each space makes sure the encoding stays close to its prior distribution.

Also based on variational autoencoders, CCVAE [117] aims to get beyond local sequence features by taking into account for binning not only the contig itself, but also the reads composing it. To do this, they use the assembly graph where nodes are the contigs and edges the k-mers connecting contigs, with a weight equal to the number of times this k-mer occurs in the data. This graph constrains the VAE to represent nodes with edges between them with more similar features. Considering this graph allows this method to outperform VAMB, and paves the way to graph embedding methods in metagenomic binning.

Finally, another method outperforming VAMB is SemiBin [118], which follows the concept of semi-supervised learning, by adding information from reference databases while still being able to discover new bins outside of reference datasets. SemiBin relies on the notion of constraints by creating must-link and cannot-link constraints between contigs. The must-link constraints are created by breaking contigs up, while the cannot-link constraints use reference contig annotations. These constraints are combined with the same inputs as VAMB (abundance by mapping and k-mer frequencies). Deep Siamese networks embed these features in a distance between two contigs, generating a sparse graph clustered with a k-means algorithm. SemiBin outperforms existing binners, in particular VAMB and SolidBin [119], on both real and simulated datasets. More specifically, it recovers with great completeness a high number of complex bins. It is precise enough to differentiate *Bacteroides vulgatus* from human and dog gut microbiomes. However, it must be noted that it uses single-copy marker genes as input to predict seed contigs, but also to validate the quality of the bin. This may artificially enhance the quality of the bin, as this quality is assessed by searching for the gene that was actually used for binning.

Of note, these binning methods work with contigs rather than raw reads. Contigs must first be generated with an independent software [120]. SemiBin demonstrates the importance of background knowledge, showing the importance of continuous database progression in the binning task. To date, sequence-composition and feature abundance methods provide the most convincing results for this kind of task, but other tools use different approaches based on promising new architectures.

### Methods inspired by natural language processing

As NLP was used for functional annotation, it is also increasingly used to classify reads and perform binning, or even analyse a metagenome.

DeepMicrobes [121] highlighted the importance of k-mer embedding, comparing this method to one-hot encoding but also introducing attention in metagenomic analysis by presenting an architecture using LSTM and self-attention-based models. The results show that embeddings significantly improve performance when compared to one-hot encoding.

Given the analogy between NLP and DNA analyses, it is not surprising to see adaptations of word embedding algorithms to DNA sequence data. The word2vec method [122] has been adapted to generate k-mer and sequence embeddings by both NLP-MeTaxa [123] and FastDNA [124]. FastDNA was reused within the Metagenome2Vec method [125] to combine word embeddings with taxonomy and create a metagenome embedding. In the context of Metagenome2Vec, the term end-to-end implies that the method encompasses the full spectrum of processes needed to convert raw metagenomic data into valuable vector representations. Meta1D-CNN tries to enhance the precision in sequence classification with NLP methods by introducing 1D-CNN. They train a word2vec algorithm with different k-mer lengths from 1 to 8 (8 giving the best results). The embedding of a sequence is obtained by calculating the mean of all k-mer embeddings and classified through convolution layers.

While these methods are proof of concepts, they have not outperformed alignment-based methods outlined earlier. These DL approaches have provided insights on the limitations or difficulties with the NLP approach. First, the amount of noise in the data must be considered, particularly here, where sequence representation is the heart of the work. Second, the comparison of genomic reads to text does not fully hold up due to the intrinsic differences between k-mers and words. K-mers not only overlap but also form a finite, known and extremely dense vocabulary, particularly for a smaller value of *k*. Furthermore, a larger *k* value results in more accurate classification as the number of distinguishing k-mers becomes increasingly prevalent. A significant limitation of this approach is that each increment of 1 in the value of *k* quadruples the size of the vocabulary. Consequently, this exponential increase leads to substantially higher computational demands.

Several ideas have been explored to solve the issue of increasing computation time with longer k-mers. One is to enlarge the vocabulary by taking longer k-mers, but regrouping some of them based on proximity criteria. META 2 [126] regroups k-mers using hash embedding or local sensitivity hashing. Reads falling in the same bucket share the same embedding. On the other hand, fastDNA has been enhanced with BRUME [127]. The idea here is that k-mers that are always present or absent together in the same reads should be considered as having the same importance in sequence embedding. Therefore, they can be grouped together, using methods such as de Bruijn graphs. The drawback is that some k-mers present in new sequences to be analysed may not have been seen by the network during training and have no embedding, and this becomes more likely as *k* grows. This methodology facilitates analyses with *k* values exceeding 30, a value made possible as the quantity of de Bruijn contigs tends to plateau. The increase in *k* value enhances the effectiveness of this method, thereby leading to better results.

These ideas open the way to new methods in metagenomic binning using more recent NLP methods such as BERT [128] and its successors. Several studies have attempted to adapt the BERT method to metagenomics, but because these models are computationally expensive, they have not gone as far as they could to produce usable results. Bi-Meta [129] adapts various NLP techniques [latent Dirichlet analysis (LDA) or latent semantic analysis (LSA)] or models (Word2Vec and a very small version of BERT), while BERTax [130] also tries to train a small BERT model to perform taxonomic classification of sequences. It reproduces the masking process but uses non-overlapping words instead of k-mers. The results of these models show that although BERT is a very powerful model, especially in detecting sequences that are not closely related, it is still limited by both its computational cost and the large diversity of microbiomes. This diversity is not yet well represented by the available data that these models would need for pre-training to achieve better performance.

A recap of methods dealing with sequence grouping is shown in Table 1, and some performance comparisons can be found in Tables S1–S5.

**Table 1.** Different DL-based methods for sequence classification This table summarizes the different methods analysing metagenomic sequences, by the articles they are described in, their names, main objectives, types of DL models and methods used, data input and release date.

Reference	Name	Objective	DL model	Input	Method	Date
[83]	DeepARG	Predicting genes in metagenomic fragments	MLP	Raw reads	Annotations	June 23, 2018
[136]	Meta-MFDL	Predicting ORFs in metagenomic fragments	MLP	Raw reads	ORF features	November 8, 2017
[81]	ONN	Predicting genes in metagenomic fragments	Ontology-aware neural network	Raw reads+phylogeny	Taxonomy	January 4, 2022
[133]	cNODE	Predicting composition from species collection	MLP	Species collection	Co-presence	March, 2022
[102]	Seq2species	Sequence taxonomic classification	CNN	Raw reads (16S RNA)	One-hot encoding	August 10, 2019
[60]	DeephageTP	Identifying phage-specific proteins	CNN	Raw reads	One-hot encoding	June 8, 2022
[82]	PlasGUN	Predicting genes in metagenomic fragments	CNN	Raw reads	One-hot encoding	May 1, 2020
[83]	PPR-Meta	Phage and plasmic detection	CNN	Raw reads (genes+proteins)	One-hot encoding	June 1, 2019
[103]	GeNet	Sequence taxonomic classification	CNN	Raw reads	One-hot encoding+rank tree	February 1, 2019
[109]	DERSI	Sequence identification	CNN	Raw reads (16S RNA)	One-hot encoding and distance between reads computing	June 24, 2022
[56]	CNN-MGP	Predicting genes in metagenomic fragments	CNN	Raw reads	Separation by GC content, then one-hot encoding	December 27, 2018
[89]	MetaMLP	Metagenome profiling	Word embedding+MLP	Raw reads	Fast sequence embedding and MLP	November 16, 2021
[85]	DeepVirFinder	Viral classification	CNN	Raw reads	Sequence encoding	October 14, 2019
[86]	RNN-VirSeeker	Viral classification	LSTM	Raw reads	One-hot encoding	December 14, 2020
[87]	CHEER	Viral classification	CNN	Raw reads	Hierarchical classification with one-hot encoding or k-mer embedding	May, 2021
[91]	VirSearcher	Viral classification	Word embedding+CNN	Raw reads+hit ratio	Word embedding+hit ratio	March 22, 2022
[90]	DETIRE	Viral classification	GCN+CNN+LSTM	Raw reads	Graph k-mer embedding+one hot encoding	June 16, 2023
[95]	DLMeta	Viral classification	CNN+Transformer	Raw reads	Local and global features	December, 2022
[202]	ViBE	Viral classification	BERT	Raw reads	Hierarchical model	July 18, 2022
[92]	VirNet	Viral classification	Attention	Raw reads	Deep attention model	December, 2018
[121]	DeepMicrobes	Sequence taxonomic classification	NLP+LSTM+attention	Raw reads (short+long)	One-hot encoding or k-mer embedding	February 4, 2020
[203]	*No found name*	Unsupervised binning	Autoencoder	Genomic fragments	Nucleotide mapping+feature extraction+tSNE+denoising	2017
[111]	MetaDEC	Unsupervised binning	Autoencoder+Adversarial Network	Raw reads	Groups reads by overlap and builds representative	May 26, 2022
[112]	*No found name*	Unsupervised binning through dimensionality reduction	Autoencoder	Contigs extracted from genomes	K-mer abundance	March 14, 2021
[130]	BERTax	Sequence taxonomic classification	BERT	Raw reads	Direct or hierarchical model	2021
[204]	*No found name*	Sequence taxonomic classification	CNN	Raw reads	A network for different lengths, k-mer count	September, 2019
[106]	*No found name*	Sequence taxonomic classification	CNN/deep belief network	Raw reads (16S RNA)	K-mer abundance	July 9, 2018
[104]	ART	Unsupervised binning	MLP	Metagenomic fragments	Naive Bayes+K mer abundance	18–23 July 2010
[100]	CNN-RAI	Sequence taxonomic classification	CNN	Raw reads	Relative abundance frequency+k mer distribution	May, 2021
[205]	MetaVelvet-DL	Metagenome assembly	CNN+LSTM	Raw reads	De Bruijn graphs and hashing	June 02, 2021
[67]	VAMB	Unsupervised binning	Variational autoencoder	Contigs of raw reads	TNF+abundance	May, 2021
[116]	AAMB	Unsupervised binning	Adversarial variational autoencoders	Contigs of raw reads	TNF+abundance	2023
[114]	CLMB	Unsupervised binning	Variational autoencoders+contrastive learning	Contigs of raw reads	TNF+abundance, noise addition and contrastive learning	November 15, 2021
[117]	CCVAE	Unsupervised binning	Variational autoencoder	Contigs of raw reads	TNF+abundance+contig structure	April 24, 2023
[206]	CoCoNet	viral metagenome binning	Dense+CNN+Siamese network	Contigs of raw reads	Fragmentation of contigs, k-mer abundance and coverage features	April 2, 2021
[118]	SemiBin	Unsupervised binning	Siamese autoencoder	Contigs of raw reads and single-copy marker genes	Computes constraints and distances between contigs	April 28, 2022
[129]	BiMeta	Unsupervised binning	NLP features+transformers	Raw reads	Replace k-mer frequency embedding by NLP embedding	October 27, 2021
[123]	NLP-MeTaxa	Sequence taxonomic classification	NLP	Raw reads	Word NLP embedding	January 23, 2021
[126]	META 2	Sequence taxonomic classification	MLP+deepset or attention	Raw reads	K-mer embedding and hashing	February 10, 2020
[124]	fastDNA	Embedding of sequence (+binning)	NLP (based on FastText)	Raw reads	K-mer embeddings→sequence embeddings	June 26, 2019
[127]	Brume	Embedding of sequence (+binning)	NLP (based on FastText)	Raw reads	K-mer hashing+k-mer embeddings→sequence embeddings	March 8, 2020

### Phenotype classification

Several proofs of concepts exist for diagnosing disease from metagenomic data with ML, thanks to algorithms like MetAML [131], Predomics [132] or SIAMCAT [133]. Diseases are not the only characteristic which can be inferred from metagenomic data: VirNet [92] for example does not perform disease detection, but tries to predict an individual’s age from their microbiome using Deep Neural Networks(DNN). This demonstrates the richness of applications of metagenomic data. Most often, what is used to classify phenotypes are abundance tables of different taxa obtained after binning. They are usually tables where the rows represent the samples examined and the columns represent the taxonomic abundances.

Metagenomic abundance data are sparse, and the number of features greatly exceeds the number of samples, making it challenging to train models that do not overfit. There are several solutions to this problem including data augmentation [134].

#### Data augmentation

Despite lowering costs in sequencing data over the past decade, data accessibility still remains an issue, particularly with regard to the availability of metadata (especially clinical patient information). Besides real data, it is also possible to simulate metagenomic data using simulators such as CAMISIM [101].

Some DL-based approaches for data augmentation exist at the abundance level, for instance by generating new samples using conditional generative adversarial networks (CGANs) [135]. The idea behind a GAN is to use two competing networks: one to generate data coherent with the input dataset, and the other to try to detect whether that dataset is real or generated. The two models are trained in an adversarial way. CGANs offer the possibility to parameterize this generation: the network can then decide to generate, for example, healthy or disease-related data. However, the issue with GAN is that finding an optimal model is often challenging, and therefore there is a risk of generating unrealistic data. Furthermore, their training requires a large amount of data. Although the proof of concept is promising, it is still a problem to get sufficient quality data to train GANs and subsequently classification models.

Variational autoencoders can also be used to generate new data [136]. They use the probability distribution of the input data to generate new modified samples. Methods such as MetaNN [137] show that it is possible to achieve better classification results compared with classic ML methods using simple NNs and data augmentation.

A problem related to data accessibility is that of unbalanced classes. This problem can be mitigated by oversampling [138], or resampling the poorly represented classes until they all have as many samples as the best represented classes, or reweighting each class and training the classifier in a one-versus-all fashion for each of them [139]. Some methods test the limits of such tools, for example by trying to build a multi-class classifier of 19 diseases from five different body sites [139]. Using class weighting and resampling, it achieves interesting results when considering the top three or five predictions and not only top one, despite a highly diverse dataset.

However, note that these methods only partially mitigate the problem of lack of data: generating new data or resampling will hardly produce samples with enough diversity to allow the model to capture the full complexity of real datasets, thus leading to overfitting with very good performance on the simulated training data but poor generalization.

#### Abundance-based approaches

##### Learning new representations

To deal with the issue of high number of features in metagenomic data, many methods use dimensionality reduction techniques. These methods consist in representing very sparse data in a smaller dimension, reducing the imbalance observed before. It is possible to use different feature selection methods as well as DL-based data transformation methods.

##### Mathematical transformations and feature selection

Different normalization methods can be combined with autoencoders to extract features [134,136, 140] from abundance data that are easier to use by DL. As an example, the ridge regression algorithm was used on gene family abundance to create lower dimension data to be analysed with a CNN [141].

While most data preprocessing methods use normalization or distribution algorithms on input tables, another method bypasses the DL training step by directly using statistical binning methods such as equal frequency binning or linear discriminant analysis, and k-means clustering after that. This work directly bins metagenomes and associates them with the correct disease, achieving good prediction accuracy [142].

##### Reducing dimension through autoencoders

Since the extraction of relevant features is a specificity of DL, different types of NN have also been used to obtain better representations and embeddings. The main issue encountered with feature selection is the loss of potentially important information. It is therefore of great importance to find efficient dimensionality reduction methods. Autoencoders are an interesting hypothesis offered by DL for relevant task-adapted dimensionality reduction [143]. Such architecture is well suited to deal with the problem of sparse matrices and low sample number. Moreover, training of the autoencoder causes the data reduction method to be adapted to the specific structure of the data.

However, the best type of autoencoder to use remains an open research area. For example, DeepMicro [66] chooses to train different types of autoencoders to find the one that extracts the most significant information for disease prediction from metagenomic data. Sparse autoencoders (SAEs), denoising autoencoders (DAEs), convolutional autoencoders (CAEs) and variational autoencoders (VAEs) were all tested and gave good results, none of them outperforming the others, the best method not being the same depending on which of six different diseases it was tested on.

Ensdeepdp takes these specificities into account by using ensemble learning to get the best possible representation [144]. The distance vector between the original metagenome in input and the reconstructed one in output acts as a disease score. This experiment is repeated with many autoencoders, VAEs and CAEs, with different architectures and parameters. The *k* best models are then selected. When analysing a new metagenome, a matrix composed of the input data and the *k* best models’ representations of thse input data are computed, thus enriching the original feature space with the most interesting representations.

##### Pretrained matrices of metagenome embedding

Some methods propose pretrained tools that rely on NLP mechanisms to generate embedding matrices that can then be reused with new data. Once the matrix of embeddings is created, the new data are simply multiplied by the embedding matrix to produce a new table of embedded data. GMEmbeddings [145] provides embeddings based on GloVe [146], an NLP algorithm, by aligning requested samples to known amplicon sequence variants (ASVs) using blast. This same GloVe algorithm can generate an embedding of a user-uploaded abundance matrix [147]. The newly created data embeddings can subsequently be categorized using traditional ML algorithms, such as random forest.

### Sequence-based approaches

#### Sequence embeddings

While most phenotype prediction methods rely on taxonomy and abundance, some use other sequence-based features. They learn embeddings of relevant sequences to classify directly with them, or to enrich abundance and composition data. These approaches have the great advantage of being ‘end-to-end’, they can avoid the computational cost of binning methods, alignment-free or not, or use binning as an auxiliary source of information.

We have already emphasized the efficiency of k-mer distribution analysis for binning. K-mer distribution also proves useful for prediction. MicroPheno [148] is based on the k-mer distribution of shallow sub-samples of 16S RNA sequences. A bootstrapping framework selects relevant sequences before computing k-mer representations, allowing classification and visualization of important sequences. Aggregation of these representations allows phenotype prediction. However, the problem with such aggregation is the loss of information over microbial interactions. K-mer distribution-based embedding is compared to other methods using learnt embeddings [149], discovered using the NeuroSEED framework [150], which uses an autoencoder to compute the distance between sequences. This allows us to represent each sequence in a latent space when compared to each other.

However, instead of the distance between sequences, another analogy can be considered for metagenomic data. This analogy is that of natural language and its connection to the language of DNA. K-mers are compared to words, sequences to sentences, and metagenomes to books in order to adapt word integration architectures to the task. In a similar way that read embeddings can be used for sequence binning, read embeddings are here used for disease prediction. For example, IDMIL [57] uses bag-of-words TF-IDF algorithms to obtain an embedding for each k-mer. It aggregates these k-mer embeddings to get read embeddings. Using the same idea, Metagenome2Vec [125] avoids the solution of simply aggregating data, which would lead to losing precision, by using fastDNA [124]. Using fastDNA on metagenomic data, it performs both read embedding and read binning, taking into account the link between words and sentences, here with k-mers and sequences.

#### Multiple instance learning with sequence embeddings in prediction

Metagenome2Vec [125], IDMIL [57] and ‘Representation counts’ [149] use a particular DL paradigm called multiple instance learning (MIL). MIL is a supervised learning paradigm that consists of learning from labelled sets of instances, known as ‘bags’, instead of learning from individually labelled instances. Each bag is associated with a single label, and contains multiple instances [151]. The fundamental assumption in MIL is that a bag is labelled positive if at least one instance in the bag is labelled positive. If they are all negative, then the bag is labelled negative. Some methods have used this paradigm to perform phenotype classification from raw sequences instead of abundance tables. When using abundance, the information carried by a sequence is reduced to the species to which it belongs. With MIL, it is possible to represent a metagenome as a bag of sequence embeddings, thus keeping the information of the sequence. However, each metagenome contains millions of sequences, which represent a gigantic computational cost. Therefore, most of the time, not all sequences are treated, but rather groups or representatives of sequences.

**Fig. 5.**
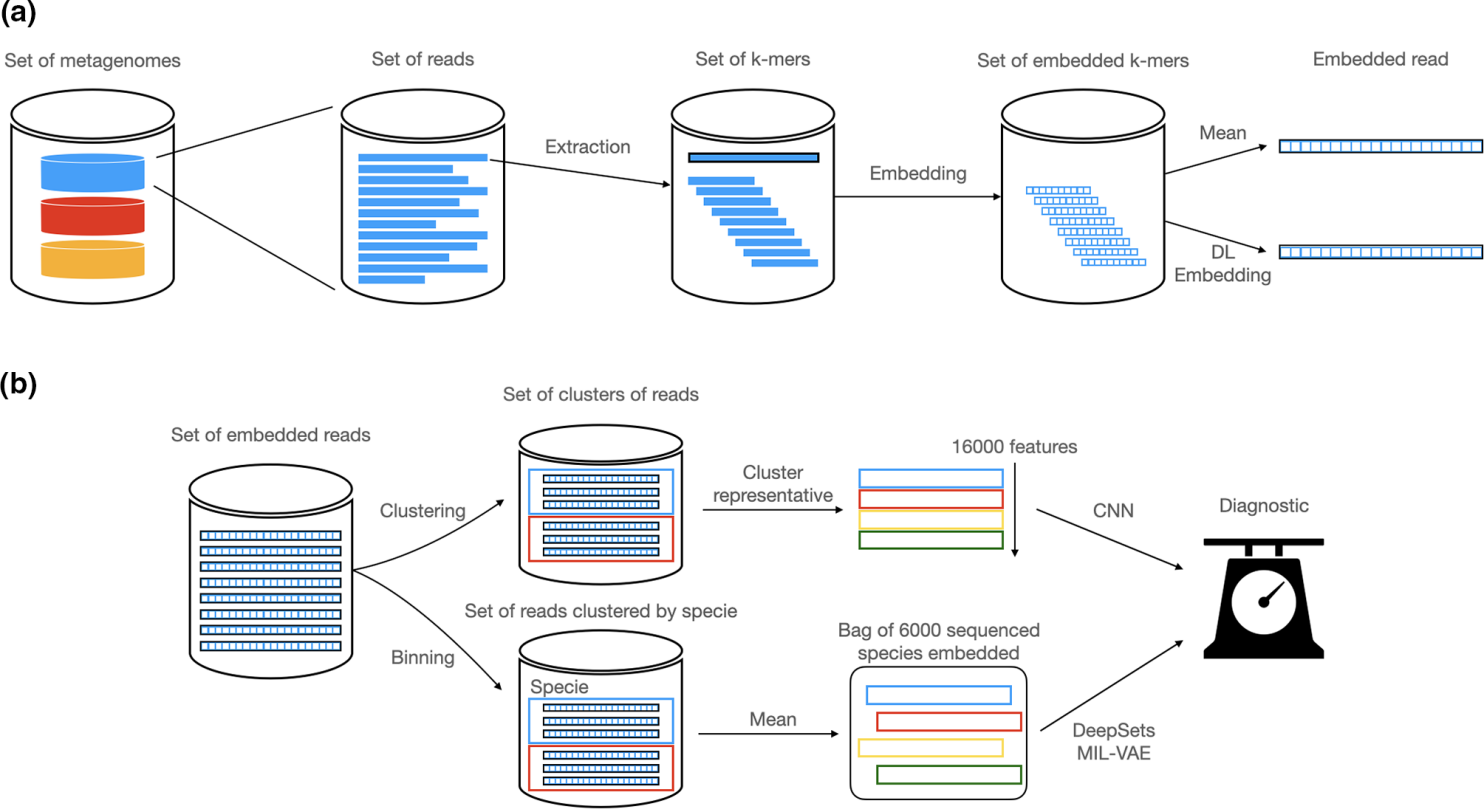
Classification with sequence embedding MIL pipelines This pipeline is shared by both Metagenome2Vec [125] and IDMIL [57]. The arrows above correspond to IDMIL, the lower ones to Metagenome2Vec. Step (a) presents how sequences are embedded: their k-mers are extracted and embedded using NLP methods. These embedded k-mers are then used to obtain the embedding of a read, whether through their mean or by learning the relationship between k-mer embeddings and read embeddings through DL. Step (b) presents how these embedded reads are grouped together. IDMIL uses unsupervised clustering with k-means, while Metagenome2Vec groups reads by genomes. Both obtain groups of read embeddings, which must then be embedded themselves. Here, IDMIL chooses a read representative for each group, while Metagenome2Vec chooses the mean. These group embeddings represent the metagenome differently: the first method orders them in a matrix and uses a CNN for prediction while Metagenome2Vec treats them like a bag of instances and uses MIL methods such as DeepSets [152] to analyse them.

In ‘Representation counts’ [149], sequences are represented through NeuroSEED. As they are obtained from 16S data, there are notably fewer sequences. They can therefore use the whole set of sequences. The problem is considered as a set classification, using all vectors and not their aggregation. To solve such a problem, they use MIL architectures like DeepSets [152] and Set Transformer [153]. IDMIL and Metagenome2Vec, on the other hand, use shotgun metagenomics data, composed of millions of sequences. The computational cost of studying millions of sequence embeddings by sample makes this idea unreasonable. However, this computational cost can be drastically reduced if instances are not sequences themselves, but groups of sequences. An example of their pipeline can be seen in Fig. 5. This is the idea followed here, with IDMIL [57] where sequences are clustered by a k-means algorithm and a representative of each cluster is used, creating ‘instances’. These instances are then ordered following their distance to a ‘centre’, computed by using the centre of the different centres of clusters. This order creates a matrix of representatives’ embeddings, which is then analysed by a CNN. An attention mechanism is also performed on these data. It allows us to differentiate and learn about the predictive interest of a given instance in the bag for metagenomic classification: which sequences are important for disease detection and which are not. However, attention being performed before the CNN, it is quite difficult to assert that it represents the true importance of each cluster. With Metagenome2Vec [125], read embeddings are clustered by species through binning using fastDNA [124] to obtain an embedding of each taxon. The metagenome is then a bag of taxon embeddings that can be analysed with MIL architectures like DeepSets and MIL-VAE. This approach is promising and end-to-end, although it still requires a binning phase. However, the way in which embeddings are exploited remains to be improved.

This paradigm, while still relatively underrepresented in contemporary literature, presents a compelling approach due to its ability to operate at a granular sequence level. This contrasts with the utilization of abundance tables, which are commonly associated with several drawbacks such as sparsity, complexities in construction, information loss and dependency on catalogues. As such, adoption of this paradigm could potentially address these challenges and enhance the precision and efficiency of ML applications in this domain.

### Integration of other types of data

Acknowledging that raw metagenomic data are not always well suited for DL, other types of data than abundance tables can be fed to give coherence to metagenomes. They are diverse and can come from the data itself or from external knowledge.

#### Taxonomy-aware learning

Abundance tables, while providing measures at the species level, do not provide information on their relative evolutionary distance. Species with close genomic sequence share similar functions and are potentially adapted to the same environment. Such information can be represented as a taxonomy tree and integrated with abundance information directly when training NNs for classification tasks. Several approaches have been tested to integrate taxonomy information: MDeep [154] groups OTUs in its vector by using a measure of correlation structure based on distance between OTUs in the tree, hoping to make phylogenetically correlated taxa close to each other. The authors then designed a CNN with three layers that are supposed to mimic the different levels of phylogeny and their interactions, with smaller numbers of neurons each time, supposedly corresponding to genus, family and order, before using dense layers. TaxoNN [155] uses a comparable yet different technique: it groups each abundance unit according to their phylum and trains a CNN for each phylum, learning the features specific to that phylum. Feature vectors from each network are then concatenated and used for final classification. The problem is then deported from species level to phylum, and phylum is analysed separately before the dense layers.

Ph-CNN [156] takes this idea further by using the distance measures in the taxonomic tree to take into account the proximity between taxa. A custom layer is designed to perform convolution on the k-nearest neighbour abundances. This method is highly dependent on the chosen distance. The drawback is that although it takes into account neighbouring taxa, it focuses on local patterns and does not process the structure of the data globally.

**Fig. 6.**
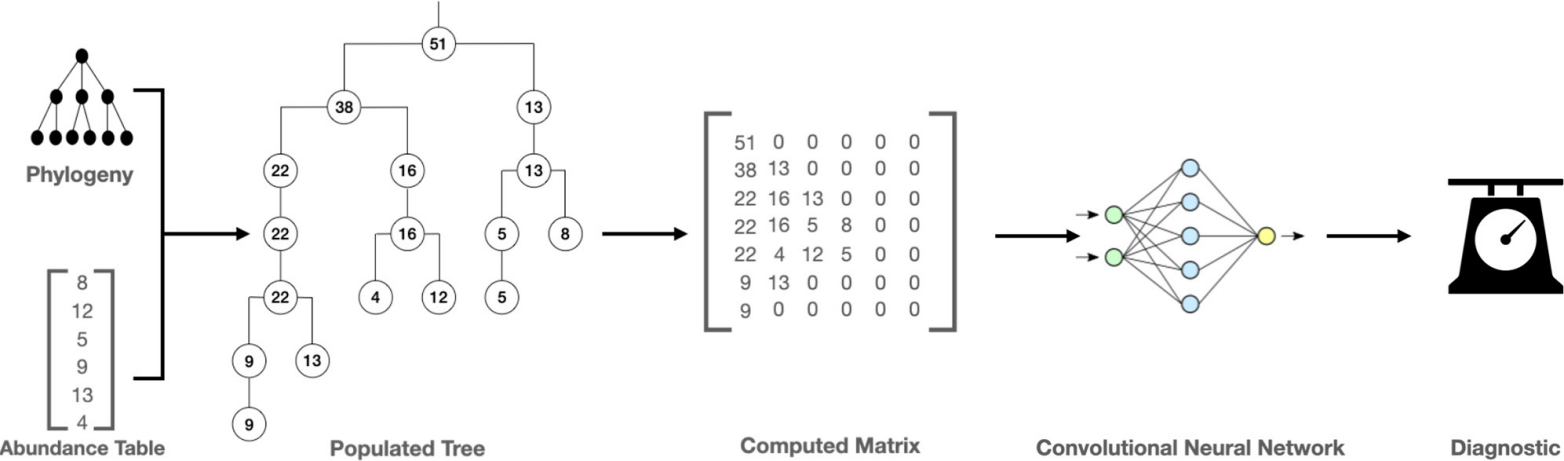
Taxonomy-aware metagenome classification method, as performed with PopPhy-CNN. Phylogeny between taxa is used to create a tree, and abundance to populate it. This tree is then embedded as a matrix used as input for a CNN that will ultimately classify the metagenome. Modified from [157].

PopPhy-CNN [157] proposes a tool that embeds the taxonomic tree in a matrix, allowing all the topological information to be processed. Fig. 6 shows the embedding algorithm chosen by PopPhy-CNN. This embedding is designed to avoid sparse matrices. The drawback of this representation is the structure of the matrix itself: embedding a tree in a matrix can result in very sparse matrices. To avoid that, this method places all nodes at the leftmost non-null spot in the matrix. A consequence is that, with a more complex tree and as nodes are placed to the leftmost spot, some nodes may not be found directly above their parents, thus blurring the links that the tree is supposed to represent. For example, in Fig. 6, the node labelled 5, found at coordinates [4, 5], is directly under the node labelled 8 [4, 4], when it is not its descendant. To consider more of the tree structure, TopoPhyCNN [158] embeds it in a matrix, but adds topological information like number of child nodes, height of layers and node distance in the tree.

These tree and graph structures present a very complex, large and potentially sparse structure. This is a serious limitation that is acknowledged by the authors, who encourage the exploration of other embedding methods. To give coherence to abundance data, some authors have tried to take spatial embedding to the level of the image: abundance data are converted and represented by an image. The Met2Img method [159] used this paradigm to outperform previous state-of-the-art tools. The abundance vector is represented as a 2D image, coloured by a taxonomy-aware fill-up method. The generated images are then analysed by a CNN to retrieve more structural metagenomic information. Furthermore, this method can be combined with the use of other omics or patient data.

Another method [160] offers direct comparison between tree-embedding methods and new image representations to show the advantages of the latter. By taking the most well-represented genera, they create different types of image representations with each genus represented by a shade of grey linked to its abundance. These images can then be analysed with a ResNet-50, a DL image analysis technique. A great advantage of this method is its interpretability, because genera that were useful for prediction of disease (here type 2 diabetes) can be easily traced. However, this method works at the genus level, at best, and by considering only the most represented genera in the data, therefore potentially omitting information coming from less well-represented bacteria.

Following the method of Met2Img, the more recent MEGMA method [161] uses manifold embedding to create a data embedding based on co-abundance patterns between microbes. Five manifold embedding methods were tested, as well as random-guided uniform embedding: MDS, LLE, ISOMAP, t-SNE and UMAP. On the other hand, microbes are grouped based on their phylogeny. This grouping will determine the colour used in the image for each group. In summary, the localization on the image is based on the embedding, while the colour is based on phylogeny, the opposite of Met2Img. This new method outperforms Met2Img and is very interpretable, and parts of the images important for prediction can be found and linked to the microbes they represent.

Finally, another aspect that can be taken into account when taxonomy is studied is the fact that a great part of it is unknown, whether it is because abundance is obtained by unsupervised binning or because reads come from unknown species. MetaDR [162] takes into account both known and unknown features as well as the topology of the taxonomy tree obtained by converting it to an image, allowing MetaDR to compete with the best state-of-the-art methods, while showing good computational speed and ranking among the best taxonomy-based methods.

#### Microbial interactions

While taxonomy offers valuable insights into the relationships between microbes, it only captures a fraction of the complex interactions within the microbiome. Microbes interact and function in myriad ways within this environment, and their taxonomic connections alone are insufficient to fully comprehend the intricate dynamics of this ecosystem. Therefore, a more holistic approach that goes beyond taxonomy is necessary to unravel the comprehensive functioning of the microbiome. This issue can be dealt with by using the abundance of each species to compute various sparse graphs of interactions between species using co-abundance patterns. The graphs are then fed into a graph embedding network designed with a specific layer for graph embedding [163]. Despite the interesting questions raised by these methods, finding other ways to analyse interactions between micro-organisms remains under-explored in the field of DL and an issue still to be addressed.

#### Functional and genetic information

Some authors have chosen to use the functions of genes or specific communities contained in a metagenome. However, as metagenomic diversity remains largely unexplored, using reference databases might be challenging or incomplete. Still, some tools try to extract relevant information from these databases. Most of these tools rely on classical ML and not DL. The method cited here [164] uses functional profiles extracted from orthologous genes given a reference database to add these features to abundance, while DeepMicro [66] uses strain-level marker profiles to contextualize and deepen abundance data by the presence or absence of a certain strain. As for abundance data, strain-level markers provide very sparse information, leading to the same difficulties. However, methods like PCA have shown satisfying results when applied on these data, leading to a slight improvement in prediction. The other way around, some ML methods aim to extract top decisive features or markers for disease prediction to understand key roles played by these features in the development of a disease [132,165].

#### Combining different sources

Using DL to try and reconcile many ways of integrating information, MDL4Microbiome [166] opens the way to adding different types of data for prediction by designing a model made of various parallel simple feed-forward NNs. Each network takes a different source of data as input and performs phenotype classification. By concatenating the last features used before classification of each network, MDL4Microbiome can obtain a vector representing each source. This model seems to outperform classical ML methods in disease classification, and shows that combining features together improves results over using each feature type separately. Here, the experiment is performed with three sources of data: species abundance, metabolic function abundance and genome-level coverage abundance, but any feature can be used following this simple model, even though its use might not be optimal.

### From cross-sectional to longitudinal metagenomics data

The human microbiome is highly dynamic and can change drastically in a short time, be it due to diseases, diet or medical interventions. All the methods described above work with single-point data. However, it is possible to study the evolution of a microbiome over time or the influence of specific events on its composition with longitudinal data, i.e. at different time steps from the same patient, for instance by analysing such data before and after dietary changes to understand their impact on the microbiome composition [135] or the transition from adenoma to cancer [167]. GraphKKE [168], on the other hand, used a DL-based approach and proposed to embed a microbiome with time-evolving graphs. Nevertheless, these methods are not strictly speaking temporal. The data are not seen as temporal series, and therefore the analyses are independent single-point analyses, and not an analysis of the evolution of the microbiome through time. The temporal study is more seen as giving coherence between different time steps and studying the longitudinal metagenomic data as a whole, rather than different time steps without linking them together.

There are other methods based on DL used to analyse real-time series data. Instead of a single point abundance vector, they consider a time series of vectors, which means a matrix containing a vector for each time step. This can be done through the use of RNNs and in particular LSTM models. These networks capture the temporal evolution of data through different time steps, for example to predict the occurrence of allergies in children aged 0 to 3 years old [169], the evolution of ulcerative colitis [61] or various diseases like type 2 diabetes, liver cirrhosis or colorectal cancer [162]. All these methods used phylogenetic information of different time steps treated as a time series by an LSTM. This has proven more effective than SVM, KNN or LR ML methods. To try and give more coherence to both each time step and their global dynamics, an approach combining CNN and LSTM was developed with phyLoSTM [170]. Here, each time step is processed following the same method as with TaxoNN [155], i.e. by ordering OTUs by phylum and using a CNN adapted for each phylum. Once the feature vector for each phylum is extracted, they are concatenated in a feature vector representing the time step. All these vectors will then form the new time series to be analysed by the LSTM. Therefore, phylogenetic information is extracted by the CNNs, while temporal features are extracted by the LSTM.

This CNN–LSTM structure has also been used with self-distillation [171,172]. Knowledge-distillation [173] is a recent and impressive NN training technique. It consists of transferring knowledge from a large and heavy model to a lighter one by training it to mimic its output. This technique saves a lot of computation time, despite a degradation in accuracy. Self-distillation consists of applying such a process to a network by itself. It is done by plugging shallow classifiers at the output of hidden layers in the network. These classifiers allow us to compare the features outputted by hidden layers to the global output of the model, and therefore teach the inner layers by the model itself. Self-distillation allowed the model to outperform many other longitudinal models [169].

MDITRE [174] performed similar work to phyLoSTM by ordering data phylogenetically and combining both spatial and temporal treatment of the data, while adding visualization with heat maps of the abundance variation over time. The authors also focused on interpretability by extracting human-readable rules that characterized the evolution of the microbiome. Some of these rules could be sentences like ‘The average abundance of selected taxa between days 118 and 183 is greater than 7 % AND the average slope of selected taxa between days 118 and 190 is greater than 0 % per day’. This helps dealing with the problem of how decisions can be taken and justified when relying on black-box models like those found in DL.

The longitudinal paradigm is particularly interesting for retrieving the emergence and progression of a disease over time. Indeed, it is not straightforward to find the causality of a disease in the microbiome using cross-sectional data, and comparing two patients with a different diagnosis is also difficult, as the differences between microbiomes may come from very different sources. Studying the same patient at different time points may allow us to reduce these sources of discrepancies while increasing the statistical power that could lead to a better understanding of the pathophysiology of the studied disease. To push the idea further, considering the best single-point analysis methods together with LSTM and other longitudinal methods might be key to understanding the most important shifts between healthy and disease states.

### The reciprocal: predicting microbiome composition

Given that a metagenome can be used to predict phenotype, one can also imagine the other way around, for example a *k* nearest-neighbour regression-based ML technique which uses species assemblage of a microbiome, i.e. their absence/presence, to recreate the abundance of each of them without needing complex interaction graphs [175]. Using DL, it is possible to infer the taxonomic composition of the original microbiome without sequencing and binning from phenotype and environmental information [176]. Similarly, G2S [177] reconstructs the composition of the stool microbiome using information from the dental microbiome: using the abundance table from the dental microbiome diversity, it generates a new abundance table supposed to represent the diversity of the stool microbiome. Finally, a method considering temporal data uses an LSTM to analyse the abundance of a given microbiome at each time step and predict the abundance of the next time step [178]. This method allows us to understand various microbiome dynamics, and can be used to understand the changes in the functions, but also the evolution in metabolite productions.

A recap of methods dealing with phenotype prediction is presented in Table 2. A performance comparison is provided in Table S6.

**Table 2.** Different tools for phenotype prediction This table summarizes the different tools studied here along with their objective, their input, their model and how they treat information. A table with links to code and dataset and additional information is visible in the Supplementary Material.

Reference	Tool	Objective	DL model	Input	Method	Date
[144]	EnsDeepDP	Phenotype classification	Autoencoder: deep, variational and convolutional	Abundance table	Ensemble learning: various data encoding, selection of the best and classification of concatenation	2022
[207]	DeepMicro	Phenotype classification	Autoencoder: shallow, deep, variational and convolutional	Abundance table+gene annotations	Learning data representation and classifying	2020
[143]	*No found name*	Phenotype classification	Autoencoder	Abundance table	Dimensionality reduction through autoencoders	2021
[140]	*No found name*	Phenotype classification	Autoencoder+NN	Abundance table	Normalization methods stacking and feature selection	2021
[137]	MetaNN	Phenotype classification	NN+CNN	Abundance table	Data augmentation	2018
[141]	*No found name*	Phenotype classification	CNN	Abundance table	Feature extraction and classification	2021
[208]	IDMIL	Phenotype classification+feature selection	CNN+embedding+attention	Raw sequences	K-mer embedding, sequence embedding, clustering and multiple instance learning	2020
[125]	Metagenome2Vec	Phenotype classification	NLP+DeepSets	Raw reads	Read embedding→genome embedding and multiple instance learning	2020
[149]	*No found name*	Phenotype classification	Autoencoder+CNN+DeepSet+transformer	Set of sequences	Set of sequence embedding: k-mer vs learnt	2022
[145]	GMEmbeddings	Metagenome embedding	NLP	Abundance table	Word embedding techniques (GloVe), PCA	2022
[148]	MicroPheno	Phenotype classification+body site identification	NN	Raw reads	K-mer distribution in shallow sub-samples	2018
[209]	*No found name*	Body site identification	NN	Raw reads	Read encoding	2019
[166]	MML4Microbiome	Phenotype classification	NN	Abundance table+gene annotations+preprocessed raw sequences	Using a network for each type of data and concatenating	2022
[154]	MDeep	Phenotype classification	CNN	Abundance table+taxon annotation	Phylogenetic distance	2021
[155]	TaxoNN	Phenotype classification	CNN	Abundance table	A CNN by phylum	2020
[158]	TopoPhyCNN	Phenotype classification	CNN	Abundance table+taxon annotation	Tree embedding and topology	2021
[157]	PopPhyCNN	Phenotype classification	CNN	Abundance table+taxon annotation	Tree embedding	2020
[156]	Ph-CNN	Phenotype classification+feature selection	CNN	Abundance table	Tree distance between OTU+CNN	2017
[163]	GEDFN	Phenotype classification+feature selection	Graph embedding Deep Feed-Forward network	Abundance table	Constructing microbe interaction graph from abundance	2019
[162]	MetaDR	Phenotype classification	CNN	Abundance table	Phylogeny mapping of abundance from known and unknown samples	2022
[139]	*No found name*	Phenotype classification	Graph convolutional Network	Abundance table	Multiclass on very big dataset	2019
[161]	MEGMA	Phenotype classification+feature selection	CNN	Abundance table	Mapping abundance to an image with manifold embedding	2023
[159,210]	Met2Img	Phenotype classification+feature selection	CNN	Abundance table	Mapping abundance to an image	2020
[160]	*No found name*	Phenotype classification+feature selection	ResNet-50 (CNN)	Abundance table	Mapping abundance to an image	2023
[211]	Meta-Signer	Phenotype classification+feature selection	NN	Abundance table	NN-based classification+rank aggregation	2021
[212]	MegaD	Phenotype classification	NN	Abundance table	NN-based classification	2022
[213]	*No found name*	Phenotype classification	MLP+RNN	Abundance table	Structure learning and classification	2015
[214]	*No found name*	Phenotype classification	NN	Abundance table	NN-based classification	2020
[215]	*No found name*	Age prediction from metagenome	NN	Abundance table	NN-based classification	2020
[165]	*No found name*	Find T2D-related biomarkers and their interactions	NN	Abundance table+gene annotations	NN-based regression for markers’ identification and interactions	2022
[174]	MDITRE	Phenotype classification through time+feature selection and data visualization	NN	Longitudinal abundance table+phylogenetic tree	Various custom layers to extract each type of feature	2021
[170]	PhyLoSTM	Phenotype classification through time	CNN+LSTM	Longitudinal abundance table+phylogenetic tree	A CNN by phylum+LSTM for temporal analysis	2023
[171]	*No found name*	Phenotype classification through time	CNN+LSTM+self-distillation	Longitudinal abundance table	CNN-LSTM+self-distillation knowledge	2023
[162]	Meta_GRU	Phenotype classification through time	RNN - GRU	Longitudinal abundance table	Feature extraction and classification	2021
[61]	*No found name*	Phenotype classification through time	Autoencoder+CNN+LSTM	Longitudinal abundance table	Feature extraction and classification	2021
[169]	*No found name*	Phenotype classification	Autoencoder+LSTM	Longitudinal abundance table	Feature extraction with autoencoder and classification	2019
[135]	*No found name*	Phenotype classification through time	Autoencoder+NN	Longitudinal abundance table	NN-based classification	2019
[142]	*No found name*	Phenotype classification	Binning techniques	Abundance table	Data transformation and clustering	2022
[177]	G2S	Predicting stool microbiome from oral microbiome	CNN	Abundance table	Rescaling and confusion matrix correction after CNN	2021
[135]	*No found name*	Simulating microbiome	Conditional GAN	Abundance table	Parametrization of new data	2020
[176]	*No found name*	Reconstruction and prediction of microbiome composition	Autoencoder +NN	Abundance table+environmental features	Reconstruction through autoencoders	2020
[178]	*No found name*	Prediction of microbiome evolution	LSTM	Abundance table	Dynamics prediction	2021

## Discussion

Despite the promising results of the methods presented here, several questions and limitations must be raised. It is important to note that these methods are not used for diagnosis in field medicine and are currently at the stage of proof of concept or feasibility studies. Recent research has shown that the complexity and diversity of real life data can be a real challenge for DL models, even if they have shown very interesting results based on data used in research [7]. This is a serious concern at a time when AI is becoming more prevalent in our society, because leaving diagnosis to a program that appears to work well but actually produces false negatives or false positives can be the cause of major health problems. The ‘black box’ nature of most DL models increases these risks by making it difficult for the human mind to understand why such a diagnosis was made. Therefore, we would like to emphasize the importance of developing explainable DL models that are trained on diverse, complete data from multiple sources and validated on independent holdout datasets. In clinical care such models, including those that use ML and classic bioinformatics processing, should be evaluated extensively similarly to drugs before they are approved by relevant authorities. Indeed, several reasons may explain the limitations encountered here.

Metagenomic data are complex, and difficulties can arise at several levels. First, generating the raw data requires complex and expensive (although becoming less so) wet lab protocols. Samples must be extracted from their environment of origin, whether natural or host, and sample collection methods, DNA extraction kits, and library preparation protocols, along with the sequencing technology of choice, are susceptible to introducing systemic biases that can affect how well the recovered microbiome profile reflects the true microbiome composition [179,181]. Also, in the bioinformatic processing of metagenomic sequencing data, the reference databases used to generate the quantitative metagenomic profiles play a key role in the accuracy of the profiles relative to the actual microbiome composition, and may introduce systematic biases if members of the microbial community under study are not well represented in the database of choice [182,183]. Finally, the choices made to handle the specific nature of metagenomic data in terms of compositionality by means of different data transformations [184,185], the use of different approaches to estimate absolute abundances of metagenomic features [186] or the different strategies that can be adopted to handle variations in sequencing depth [187,188] have a major impact on the quantitative profiles that will be subsequently used for biomarker discovery or phenotype prediction [189,190]. Although standardized protocols [191,192] and integrated reference repositories of quantitative metagenomics datasets [193,194] have been proposed to mitigate these biases, the importance of these steps need to be underlined, as they are the source of all data used to train the models, and learning from corrupted data will hardly lead to reliable predictions. This has recently been highlighted in the polemic surrounding the predictive power of microbial DNA derived from human tumour samples in predicting various types of human cancer, where findings from a 2020 meta-study [195] have been shown to be strongly influenced by the presence of undetected sequences of human origin that were not properly filtered out, by the inclusion of microbial species in predictive models with implausible presence in human samples as a product of the database choice in the taxonomic profiling of non-human reads, and by the adoption of a normalization strategy derived from RNA sequencing analyses that introduced errors in the transformation of the raw data, creating an artificial signature for microbes with no detected reads [196]. As stated before, metagenomic abundance data impose several constraints that DL models can have trouble handling.

These protocols, along with privacy concerns surrounding medical metagenomic samples, make these data still rare and expensive. DL models require very large datasets to train and be able to generalize to new data. To date, the number of metagenomic samples used to train most of the models presented here is only in the hundreds, at best thousands, which is below the amount needed to efficiently train DL models. Moreover, these small numbers highlight another problem: the diversity of these datasets. In fact, many factors other than disease can alter the composition of the microbiome, such as patient geography or diet. If a model is designed to detect disease, but has only been trained on data from patients of a certain type, it may not generalize well to patients of different origins or lifestyles. In this context, it has been shown that 71 % of human metagenomic datasets publicly available in sequence repositories such as the Sequence Read Archive (SRA) are from Europe, the USA (46 % of the total) and Canada (highly developed countries representing only 4.3 % of the world’s population), indicating a critical need in metagenomic studies to have a more global representation of human populations from non-developed countries [197].

The lack of diversity in these data can lead to severe overfitting and is a serious limitation. After analysing several articles, we would like to point out the lack of a solid meta-analysis of DL in metagenomics. Most developed methods compare themselves with alignment-based methods or classical ML methods such as MetaML [131], but there is a lack of comparison between DL methods, especially between methods with similar goals but different approaches. Simulated datasets from the CAMI project [101] are often used, but they are highly dependent on reference genomes and may lack diversity. The definition of evaluation metrics is also difficult: species classification, quality of bins or differentiation of closely related species can give different results. In the case of disease prediction, the datasets are very diverse and no data harmonization has been performed. Moreover, few methods use true holdout datasets and therefore evaluate their performance on validation datasets that are closely related to the training sets; sometimes they are even evaluated directly on training sets. Table S6 summarizes these different results reported by each article.

Of course, the quantity of data is of primary importance, but the type of data and the coherence between the pieces of information is just as much of an issue [166]. As we have seen, classifying a microbiome almost always means using its taxonomic abundance vector. This structure in itself contains several constraints that can be challenging for DL methods. The high number of features and sparsity can lead to overfitting, but the compositional nature can also be a difficulty: the constraint that abundances add up to 100 % can sometimes not be respected by NNs, which are sensitive to input size and may not detect relationships between the different components of the microbiome. Moreover, it must be put into perspective with the question of whether microbial communities sorted taxonomically are relevant predictors for these diseases. For a good prediction, we would need communities of micro-organisms that are associated in the same way with the studied phenotype. This would mean communities acting positively, negatively or neutrally for a disease in the same way and ‘quantity’. Taxonomic communities have many advantages, because closely related microbes have a high probability of sharing common behaviours. However, some recent studies have shown that very closely related individuals can behave very differently [198], sometimes even in opposite ways, despite their taxonomic proximity. This could lead to communities containing microbes acting both positively and negatively, making the community appear neutral. Different approaches are therefore imagined, based for example on guilds [198]. Guilds are based on co-abundance and represent organisms that act in the same direction and therefore evolve together, supposedly in the same dynamics. Questioning the way micro-organisms are grouped could be an interesting way to better characterize a metagenome and ultimately improve downstream classification tasks.

Apart from the question of datasets, understanding how the prediction is made to detect potential unexpected behaviours is key in the medical field [69]. Such models can also be useful to understand the importance or discover new biomarkers Many ML methods are quite useful for interpretability. For example, non-DL methods like MarkerML [199] allow the discovery of biomarkers but also the visualization of their interactions, while Predomics [132] explores the best signatures though very simple models to predict phenotype and allows their features to be explored. The high number of transformations and the level of abstraction induced by the layered structure of NNs obscure the way the decision was made. Extracting weights of neurons to assert their importance is one possible solution [163], but as the network grows in complexity, it becomes more difficult and unclear. To address this issue, the images created by Met2Img [159] are organized using background knowledge such as the ontology of the species. Ablation studies may then be used to identify which parts of the image are most useful to the decision and relate these parts to related species. Besides images, saliency maps can also be calculated to understand which features were mostly used for classification [200]. Time-evolving methods, by incorporating temporal data, represent a great opportunity in finding new approaches for interpretability, as they permit the extraction of correlations between changes in features and in phenotype. The rules derived using MDITRE [174] are a good step in this direction. The problem remains the fact that microbiome interactions are highly complex and non-linear, and most of these methods acknowledge the importance of each feature individually, or the comparison of two of them at most, but can hardly give any insight on larger interactions.

Especially since new powerful DL models are emerging today, we suggest that a future meta-analysis should include the probable future applications of these models in metagenomics, especially considering the development of large language models. These models produce impressive results for many tasks, and their applications in our field will certainly be of interest. Their use for other disease prediction, such as Covid 19, has already shown promising proof-of-concept results [201]. Of course, these methods remain challenging because they require very large and representative databases, while microbiomes are still composed of many unknown micro-organisms.

**Fig. 7.**
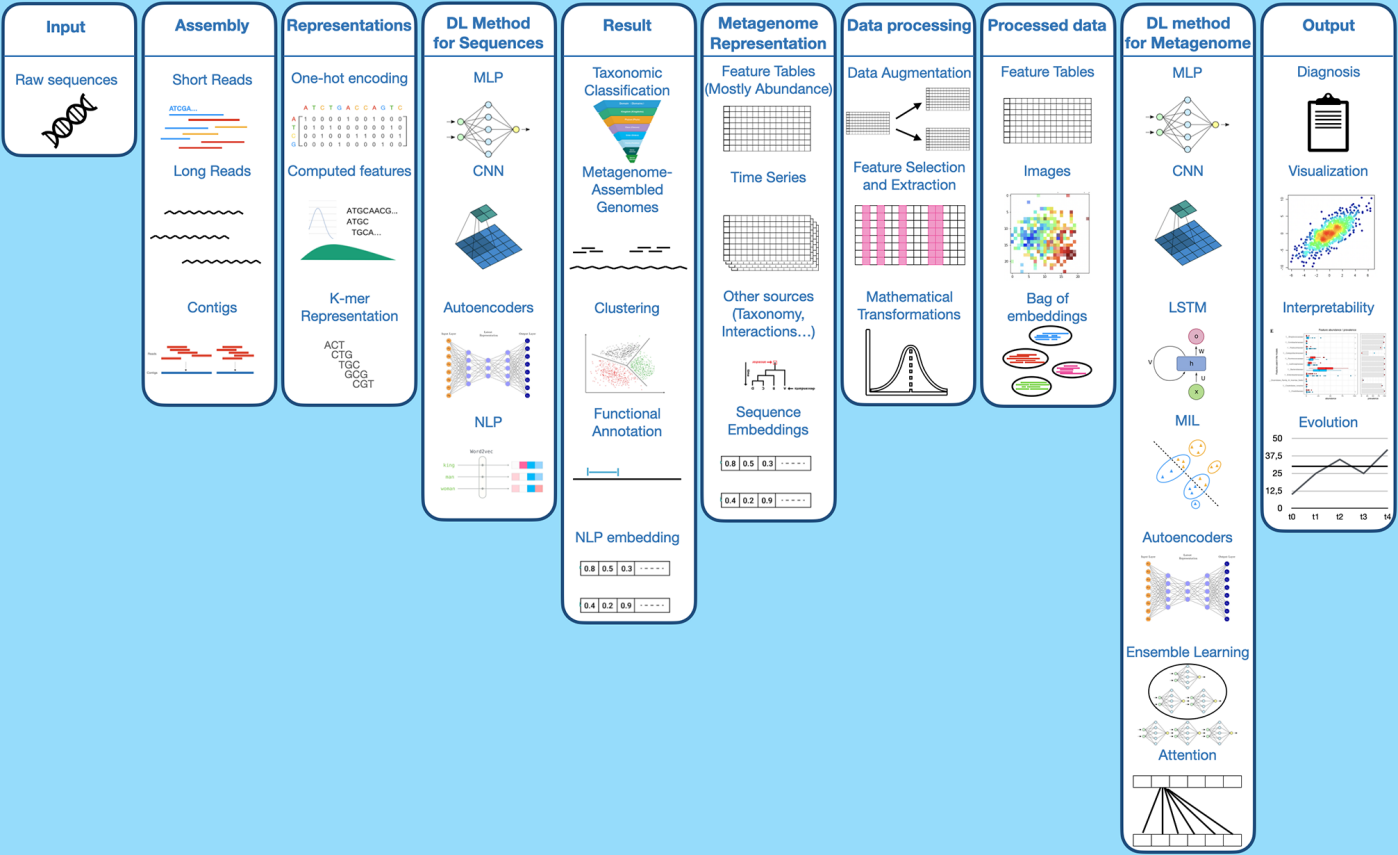
Overview of different steps and methods in disease prediction from metagenomic data. These steps represent the entire pipeline from raw reads to disease prediction. Note that not all steps are required and some methods described in a step are not always compatible with every method from the next step. This figure aims to represent the diversity of method in each step, not necessarily every entire pipeline possible. Moreover, as previously stated, most methods only perform half of the steps: the first half from reads or contigs (steps Input or Assembly) to their classification (steps Result or Metagenome Representation) and the second half for disease prediction (step Metagenome Representation to Output). Input represents the raw sequences acquired through sequencing. Assembly can either be the long or short reads acquired previously, or the contigs assembled from these reads. Representations are the way these features will be fed to the DL model (encoding, features). DL Method for Sequences show the different types of networks used to extract features. Results are the output of these networks: classification, clustering and embedding, which can then be used for Metagenome Representation, along with other sources. These representations are then filtered or transformed through Data processing, resulting in Processed data (images, tables, clusters). DL method for Metagenome are then used to treat these features and produce an Output: diagnosis, data visualization, phenotype evolution.

For this metagenomic review, we wanted to focus exclusively on the intersection between the two fields of DL and metagenomics. In need of a reproducible method, we designed a specific search equation. The objective of this equation was to select articles from all other fields while remaining stringent in order to focus on our theme, as both themes composing it present a large literature. This is why our equation is very specific and searches for words in the title, which can be considered as too stringent. We are aware of this limit, and this is why we decided to enrich our database with connected papers. We are aware that such a choice relies on external tools and leads to choices that can be considered arbitrary, such as choosing a threshold for the connectivity of articles found via connected papers. However, we considered it to be a rich source of data reproducible by anyone. It was important to have a complete overview of the field, so we chose to report together the different steps of metagenomic data analysis and their various scales (sequences, abundance tables, time series). An overview of the different steps and methods is shown in Fig. 7.

## Conclusion

DL has emerged as a promising alternative to traditional bioinformatics approaches in metagenomics in just a few years, for tasks such as binning, sequence prediction, pathogen detection and phenotype classification. Despite the promising performance of DL in metagenomics, a good understanding of the nature of metagenomic data itself remains essential. New sequencing technologies, ever-growing catalogues of species and genes, and studies of microbial interactions may require new approaches to using metagenomic data for disease prediction. Meanwhile, DL and especially powerful transformer-based models such as BERT and GPT, are rapidly advancing and offer significant potential for data analysis in metagenomics, but are still underutilized due to their high computational requirements. These models have a large number of parameters (345 million for BERT and 175 billion for GPT-3), requiring even more data to train effectively. While these data are currently difficult to obtain, their recent and rapid expansion could outpace traditional ML algorithms for prediction tasks, paving the way for new models and results. Finally, future work should focus on improving end-to-end analysis of metagenomic data to enable point-of-care applications.

## supplementary material

10.1099/mgen.0.001231 Uncited Fig. S1.

## Abbreviations

ASV: amplicon sequence variant
CAE: convolutional autoencoder
CGAN: conditional generative adversarial network
CNN: convolutional neural network
DAE: denoising autoencoder
DL: deep learning
FFN: feed-forward network
GAN: generative adversarial network
ITS: internal transcribed spacer
LSTM: long short-term memory
MAG: metagenome assembled genome
MGS: metagenomic species
MIL: multiple instance learning
ML: machine learning
MLP: multi-layer perceptron
NGS: next-generation sequencing
NLP: natural language processing
NN: neural network
RNN: recurrent neural network
SAE: sparse autoencoder
SOTA: state of the art
SVM: support vector machine
TNF: tetranucleotide frequency
VAE: variational autoencoder
WGS: whole-genome sequencing
