# Supplementary material for: Deep learning methods in metagenomics: a review
Source: Microb Genom. 2024 Apr 17;10(4):001231. doi: 10.1099/mgen.0.001231 (PMC11092122; doi:10.1099/mgen.0.001231)
Supplement: Uncited Fig. S1. [file mgen-10-01231-s001.pdf]

Supplementary Material

March 19th, 2024

1 Supplementary Data

1.1 Databases

As stated in Material and Methods, we used three databases for our researches : Google Scholar (https://scholar.google.com/), PubMed (https://pubmed. ncbi.nlm.nih.gov/) and IEEE Xplore (https://ieeexplore.ieee.org/Xplore/ home.jsp). We later enriched them with Connected papers : https://www. connectedpapers.com/ which is linked with Semantic Scholar Paper Corpus : https://www.semanticscholar.org/paper/Construction-of-the-Literature-Graph-in-Semantic-Amma649def34f8be52c8b66281af98ae884c09aef38b

All these databases were screened twice, the first time in July 2022 and the second one in July 2023.

1.2 Data Collection

For each article, we collected data on:

- the report: pdf, author, year, and source of publication
- the study: objective, sample characteristics, methods used and features extracted

- the public availability: DOI or url
- when relevant, the graph from Connected Papers to enrich our dataset

1.3 Filtering

The complete filtering process can be found in our manuscript

1.4 Article Classification

Two screenings were performed, the first one in July 2022 and the second one in July 2023. Tables S7 to S12 and Figures S1 and S3 deal with the first screening, while table S13 deal with the second one. In the first screening, during a second step, we classified manually the articles according to their main objective. This resulted in three main groups: (i) the Taxonomic Classification group with 37 articles, (ii) the Phenotype Prediction group with 49 articles and (iii) a small Miscellaneous group with 4 articles. The first two groups had two articles in common which were omitted (n = 90-2 in common = 88).

This classification was used only for the first screening.

For our synthesis, we used a tag annotation to classify different researches depending on different criteria.

- The objective pursued in the article (binning/sequence identification, disease prediction)
- The input used by the software (raw reads, contigs, abundance table)
- The features extracted from these inputs or external features (abundance, mapping, sequence encoding, k-mer embedding, k-mer distribution, data augmentation, taxonomy, denoising, hashing, co-presence, time series or use of reference database)

- The Deep Learning methods used (classic MLP, autoencoders, CNN, RNN, adversarial networks, NLP, siamese networks and graph embedding)

These tags will help in classifying articles, but have no influence over our selection. Different tags and the number of articles presenting each tag can be found in Tables 9 for goals, 11 for Deep Learning methods, 10 for inputs and 13 for different features used.

1.5 Public Availability

The availability of the articles retrieved are visible in Table S8.

1.6 Data Availability

All publicly available or open-access articles are described in Table ”Table of all studies”. Notebook and codes used to sort and filter articles and enrich via Connected Papers can be found on https://github.com/CorvusVaine/analyzing_connected_papers_articles.git

1.7 Authors

This section deals with authors participation in screening. During the screening phase, the search equation was designed by Gaspar Roy and validated by Edi Prifti. The enrichment method was jointly designed and discussed by Gaspar Roy, Edi Prifti and Jean-Daniel Zucker. Both these methods were discussed between authors in order to keep a reproducible screening method being exhaustive while stringent enough. The enrichment aimed to leave no important article behind in order to avoid strong biases that could have been induced by the research equation. Finally, the filtering was performed to make sure this enrichment did not create new biases by adding articles related to only one of the themes but not the other one. The screening and synthesis was made by Gaspar Roy under

the direction and review of Edi Prifti, Eugeni Belda and Jean-Daniel Zucker.

1.8 Table Preparation

Tables 1 and 7 have been prepared to summarize the various analyses cited in the review performing on metagenomic data. They were separated in two levels depending both on the data used and the objective pursued. The separation is therefore between work at sequence level and work at metagenomic profile level and hence between predicting the nature or origin of a sequence, and predicting a phenotype or visalizing the function of a microbiome. Inside each table, we then sorted the articles by their more accurate objective : classifying if a sequence is of a certain type, taxonomic classification, binning, or on the other side phenotype prediction, temporary data, etc.. We tried the most in this classification to sort methods depending on their DL architecture and then, finally, depending on the date they were released. These tables act as an inventory of all methods, but do not ambition to say much on their performances. We tried to do so in other tables. As we shall say in the **Discussion**, comparison between methods is still a quite open field, and is therefore not an easy task. We tried to give an idea by citing in Table 8 the different results claimed by each method depending on the predicted disease. But if there is a limited number of diseases that can be assessed from microbiome data, many datasets exist, which can be a source of biases, as many parameters can influence one's health including ethnicity or alimentation. In the same idea, many different datasets can be used for sequence analysis, as seen in table 2. However, the possibility to simulate reads with tools such as CAMISIM opens new perspectives for comparison. We took the time to make Tables 3,4,5 and 6. Indeed, VAMB set a standard in DL Binning, and most recent advances compared themselves to this tool and not only classic bioinformatics tools or Machine Learning methods. Therefore, it appeared to

us of interest to the user to have these information. It is noteworthy that the comparison to VAMB was always made by the authors of the latter method.

2 Details on our first screening

This section mostly contains details, figures, tables and statistics concerning the research method of articles. Connected Papers can be found at https://www.connectedpapers.com/ Two screenings were performed, the first one in July 2022 and the second one in July 2023. Tables S1 to S6 and Figures S1 andS3 deal with the first screening, while table S7 deal with the second one. In the first screening, during a second step, we classified manually the articles according to their main objective. This resulted in three main groups: (i) the Taxonomic Classification group with 37 articles, (ii) the Phenotype Prediction group with 49 articles and (iii) a small Miscellaneous group with 4 articles. The first two groups had two articles in common which were omitted (n = 90-2 in common = 88). We then generated a graph for each of the first two groups (respectively named G_{tc} and G_{pp}), the miscellaneous group was discarded as it contained irrelevant articles. The connectivity distribution (i.e. the number of links for each node) was higher in the Phenotype Prediction group compared to Sequence Classification, with a large number of already selected articles pointing to each other. It is possible to see different graphs of articles for different co-citation connectivity thresholds along with the distribution of articles in **Figure S3**. We decided to add to our dataset the articles with a co-citation connectivity > 4 for both graphs G_{tc} and G_{pp}. This filtering allowed us to recover 21 and 37 new articles, respectively. Using all articles together to form a graph (called G_{all}), we recovered 10 more articles with the same threshold. We chose this threshold because it allowed us to reject as few articles as possible while not adding more articles than the original database size. For example, given a total

of 114 articles, a threshold of three identifies 147 additional articles, more than doubling the dataset of articles. We therefore chose a threshold of four, which added 68 previously unseen articles. A majority of the newly discovered articles deal with the Phenotype Prediction group rather than Sequence Classification : 37 were found with G_{pp}, of which 12 (36%) were retained after filtering, 21 were found with G_{tc}, 2 after filtering (9.5%) and 68 were found considering G_{all} , with 17 conserved(25%). After filtering, these 17 supplementary articles are kept and added to the initial corpus for further analyses. No thematic sort was preformed for the second screening.

3 Supplementary Tables and Figures

3.1 Performance Tables

This section contains performance tables on different types of methods. It is to be noted that the performances shown here are giver by articles studied and not by an independant analysis.

Tables S1, S2, S3, S4 and S5 deal with sequence-based methods.

Article	Simulated Short Reads	Simulated Long Reads	Real Short Reads	Real Long Reads
[1]	F-measure : 0.8716	F-measure : 0.9595	F-measure : 0.701	/
[2]	/	/	Accuracy on genus-level : 0.761	/
[3]	/	/	F1 on species : 0.9894	/
[4]	/	/	V-Measure : 0.932	/
[5]	/	/	Species : Precision : 80 and Recall : 0.891	/
[6]	/	/	Genus Accuracy : 0.913	/
[7]	/	/	Genus Precision : 0.969 and Recall : 0.866	/
[8]	/	/	Clustering V-measure : 0.932	/
[9]	/	/	Species-level F1 : 0.907	/
[10]	/	/	/	Species Precision : 0.973 and Recall : 0.3305
[11]	/	/	Species-level on 100 genomes : 0.8864	/
[12]	/	/	Species-level Accuracy : 0.739	/
[13]	/	Accuracy : 0.783	/	/
[14]	F1 : 0.6589	/	/	/
[15]	/	/	Phylum-level Accuracy : 0.601	/
[16]	Accuracy : 0.8795	Accuracy : 0.9844	/	/

Table S 1: **Table listing different DL-based methods as well as their performance in taxonomic classification.** Please note that the results are found on each model's own dataset, not on a centralized dataset, using different metrics and data of various complexity, and can therefore not easily be compared.

Method	Airways	GI	Oral	Skin	Urog
VAMB	143	180	142	284	131
CLMB	144	201	163	253	155

Table S 2: **Table of comparison of performances in High-Quality genome recovery.** This table compares the number of genomes retrieved by VAMB ([17] and CLMB ([18]) as described in the latter article. This comparison takes place over real datasets from Airways, Gastrointestinal, Oral, Skin and Urogenital microbiome.

Method	Simulated Skin	Simulated Oral	Real Human Gut	Real Dog Gut	Real Ocean	Real Soil
VAMB	63	88	344	97	233	29
SemiBin	75	108	368	100	314	81

Table S 3: **Table of comparison of performances in High-Quality distinct species with multi-sample binning.** This table compares the number of high-quality distinct species returned with multi-sample binning by VAMB ([17] and SemiBin ([19]) as described in the latter article. This comparison takes place over simulated CAMI datasets of Skin and Oral microbiome, as well as real datasets from Human gut, dog gut, soil and ocean microbiome.

Method	Airways	Gastro-intestinal	Oral	Skin	Urogenital	Total
VAMB	63	82	124	72	78	440
AAMB	74	98	118	90	70	472
AVAMB	82	103	138	104	83	532
MetaBAT2	36	76	68	62	66	309
SemiBin	96	140	159	138	112	645

Table S 4: **Table of comparison of performances in Near-Complete Genomes reconstructed from the CAMI2 datasets.** This table compares the number of near-complete genomes reconstructed by VAMB ([17]), MetaBAT2 ([20]), SemiBin ([19]), AAMB and AVAMB ([21]) as described in the preprint of the latter method. This comparison takes place over simulated CAMI datasets of Airways, Gastro-intestinal, Oral, Skin and Urogenital microbiome.

Method	Aale	Mari	Damh	Hjor	Hade	Viby	Total
MetaBAT2	53	41	50	28	51	30	309
VAMB	42	37.3	41.3	22	47.3	19	208.9
VAE+E+SCG	60.4	47.4	49.8	27.8	52.4	29	266.2

Table S 5: **Table of comparison of performances in High-quality bins.** This table compares the number of high-quality bins reconstructed by VAMB ([17]), MetaBAT2 ([20]) and VAE+E+SCG ([22]) as described in the latter's article. This comparison takes place over Wastewater Treatment Plant datasets

Article	CRC	IBD	CIR	OBE	T2D	W2D	CrD	UC	Year	Tool
[23]	/	/	/	/	Mean AUC : 0.811	/	/	/	2022	/
[24]	0.834	0.844	0.949	0.677	0.776	0.786	/	/	2022	/
[25]	/	0.81	/	/	/	/	/	/	2022	GMEmbeddings
[26]	0.894	0.941	0.911	0.714	0.771	0.860	/	/	2022	EnsDeepDP
[27]	0.803	0.955	0.940	0.659	0.763	0.899	/	/	2020	DeepMicro
[28]	/	0.89	/	/	/	/	/	/	2019	MetaNN
[29]	0.818	0.863	0.862	0.656	0.564	0.704	/	/	2021	CNN1D
[30]	0.857 to 0.987	/	/	/	/	/	/	/	2021	/
[31]	0.895	0.882	0.951	0.793	0.816	/	/	/	2020	IDMIL
[32]	0.81	/	0.83	/	/	/	/	/	2020	Metagenome2Vec
[33]	/	/	/	/	/	/	0.884	/	2022	/
[34]	/	/	/	/	/	/	0.76	/	2018	MicroPheno
[35]	0.988	0.991	0.886	/	0.735	/	/	/	2022	MML4Microbiome
[36]	/	/	0.946	0.666	0.69	/	/	/	2020	PopPhyCNN
[37]	/	/	/	/	/	/	0.926	0.946	2018	Ph-CNN
[38]	/	/	0.938	/	0.762	/	/	/	2020	TaxoNN
[39]	/	0.843	/	/	/	/	/	/	2019	GEDFN
[40]	0.9063	/	0.9535	/	0.7890 to 0.8131	/	/	/	2022	EPCNN
[41]	Top 1 : 0.36 / Top 5 : .84	Top 1 : 0.4 / Top 5 : 0.94	/	/	/	/	/	/	2019	/
[42]	F1 : 0.549	AUC : 0.940	0.949	0.642	0.740	/	/	/	2023	MEGMA
[43] [44]	0.820	/	0.926	0.696	/	0.749	/	/	2020	Met2Img
[45]	/	/	/	/	0.96	/	/	/	2023	/
[46]	/	/	0.833	/	0.7	/	/	/	2022	MegaD
[47]	/	/	/	/	/	/	/	0.889	2023	/

Table S 6: **Table of different tools' performances in predicting various diseases.** The given score is ROC AUC, the diseases are Colorectal Cancer (CRC), Inflammatory Bowel Disease (IBD), Cirrhosis (CIR), Obesity (OBE), Type 2 Diabetes (T2D and W2D), Crohn Disease (CrD) and Ulcerative Colitis (UC). The results are found on each model's own dataset, not on a centralized dataset, which limits their comparability.

3.2 Figures

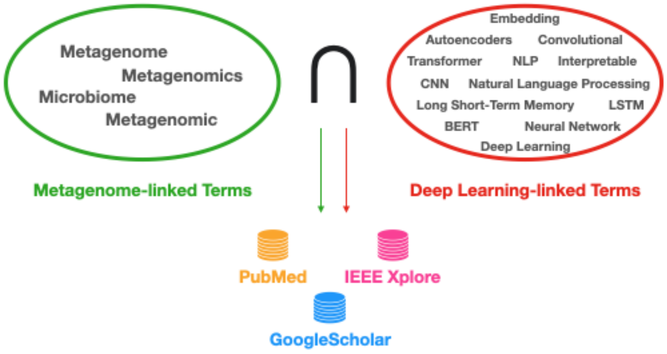

Fig S1. Diagram representing the structure of the research equation*. Each ellipse represents a part of the research equation, respectively the metagenomics (colored in green) and DL concepts (colored in red. The equation was applied to three databases: PubMed, IEEE Xplore and Google Scholar were queried.*

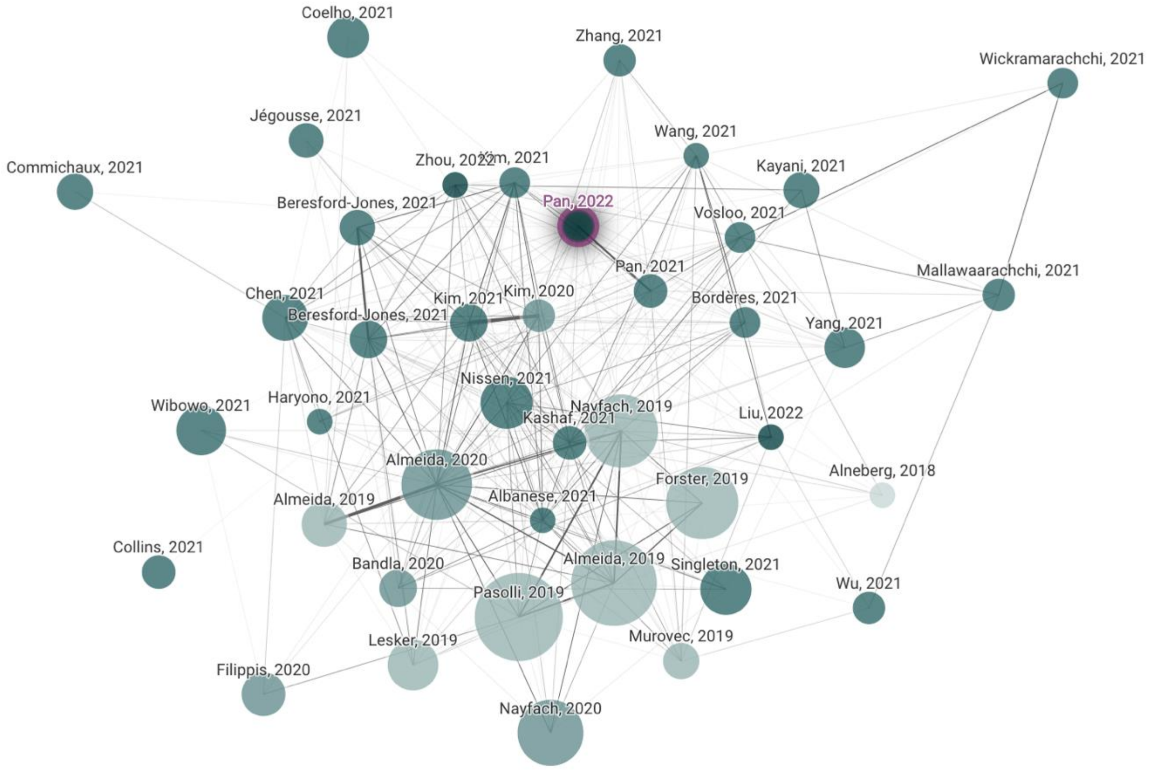

Fig S 2: This is an example of a graph generated using Connected Papers*. It has been generated from article "A deep siamese neural network improves metagenome-assembled genomes in microbiome datasets across different envi- ronments" ([19]). The darker the color is, the more recent the article, and the bigger the circle is, the most citations it has.*

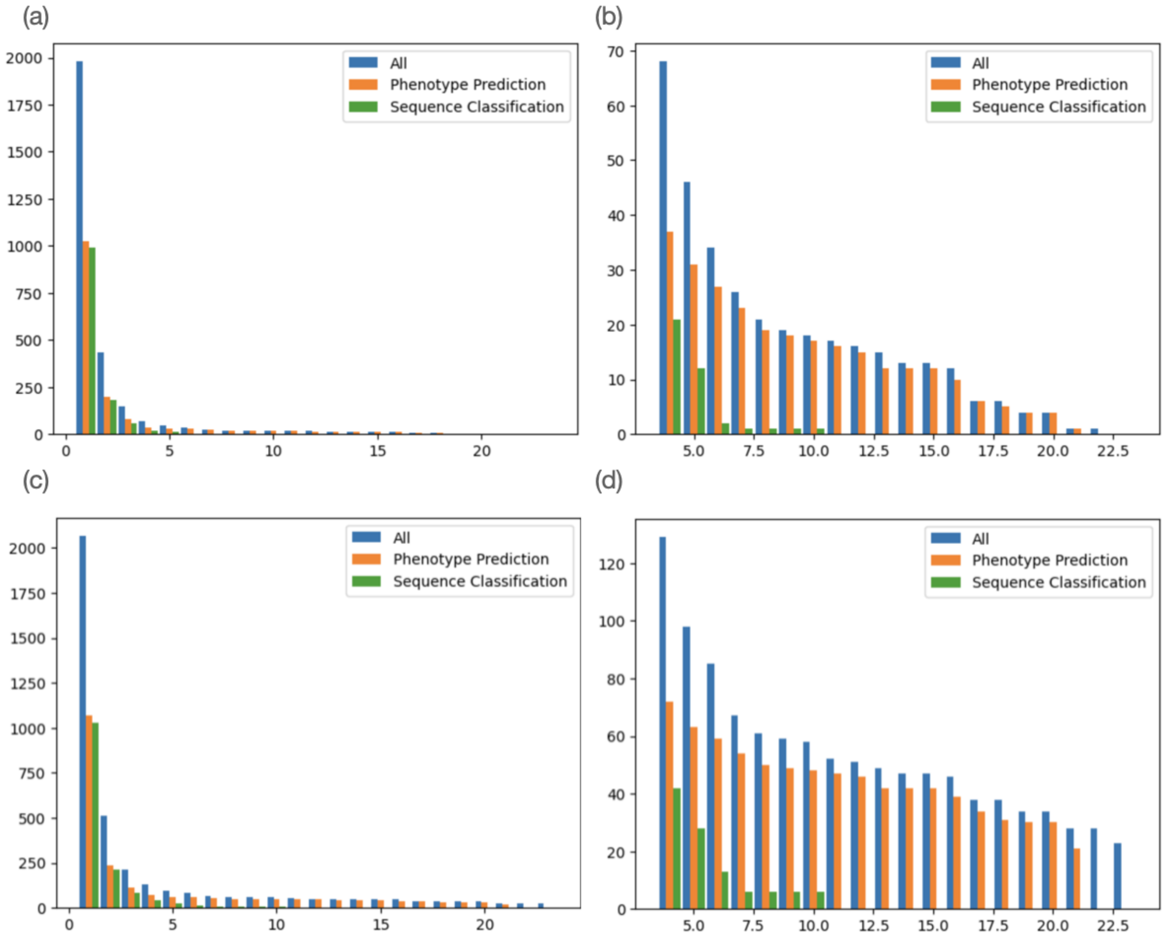

Fig S 3: This figure shows the number of articles according to the number of links from original articles pointing to them. *"Sequence classification" values mean the number of articles pointed by one of the articles of this category, same for "Phenotype prediction". "All" represents all articles pointed by those two categories + 4 miscellaneous articles.* ***(a)*** *represents the distribution of articles through the number of times they are pointed at,* ***(b)*** *is a zoom of the precedent figure, starting with a threshold of minimum 4 citations.* ***(c)*** *and* ***(d)*** *represent the same thing but considering only newly discovered articles and not the ones already present in the database.*

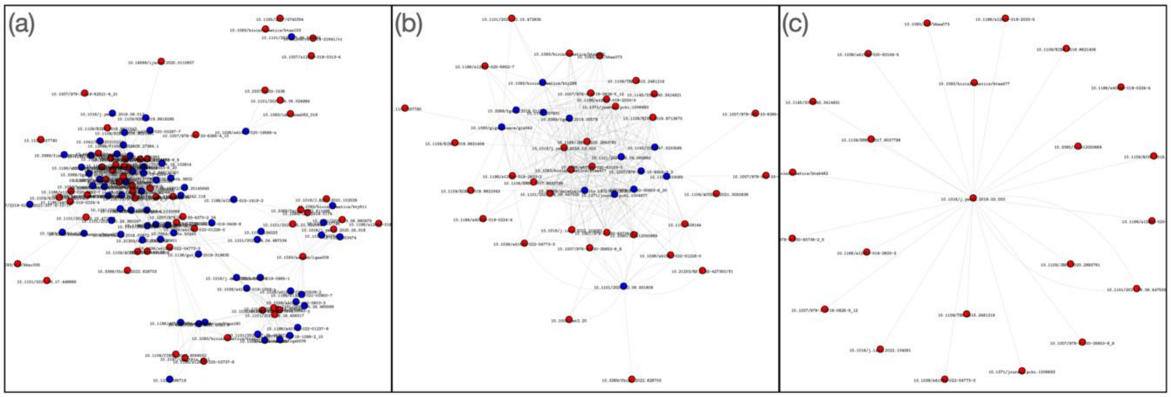

Fig S 4: Examples of generated graphs of articles : a vertex is an article (labelled by DOI). *Red dots represent articles that were already selected by research equation, blue dots represent articles discovered through Connected Papers. Only articles pointed by a certain minimal number of links defined before. These articles consider the total of all articles, not only the "Sequence classification" or "Phenotype prediction" selections. (a) represents articles pointed by at least 4 links, (b) articles pointed by at least 14 articles and (c) articles pointed by at least 23 articles. We can see in the latter that only already presented articles remain, showing our research equation already had a firm grasp on the core of our subject*

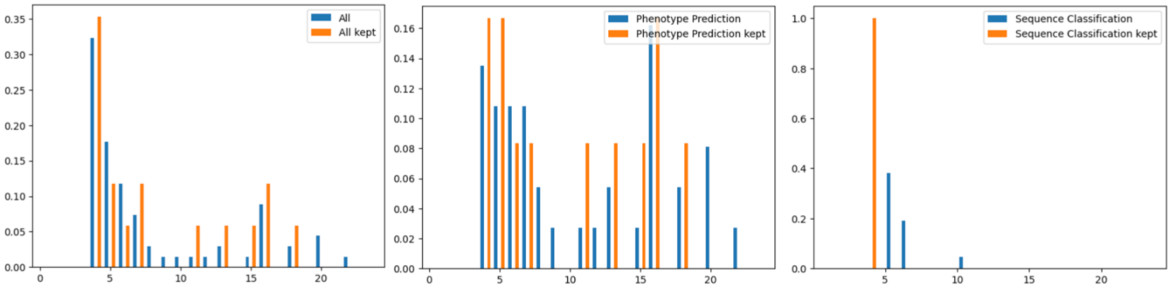

Fig S 5: The proportion of each connectivity in newly discovered articles and articles that passed the last filter

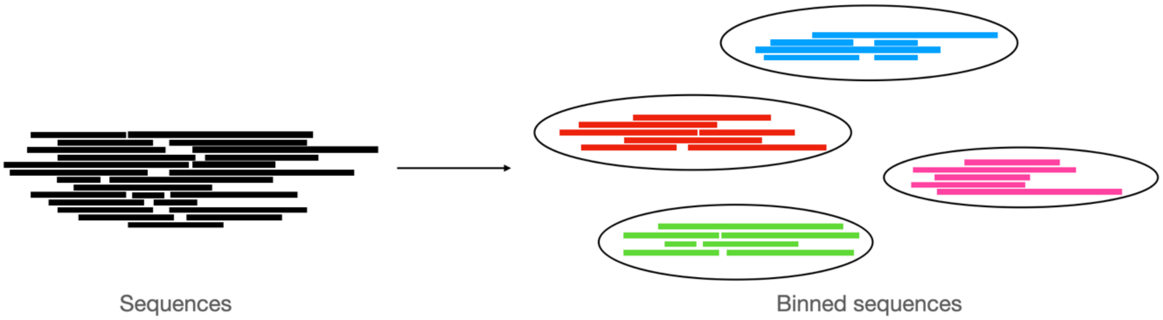

Fig S 6: Binning : grouping of sequences in different bins based on similarity

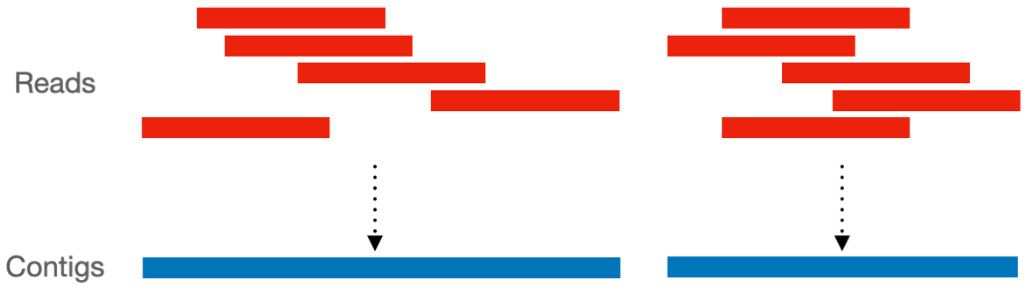

Fig S 7: Overlapping reads are combined into contigs

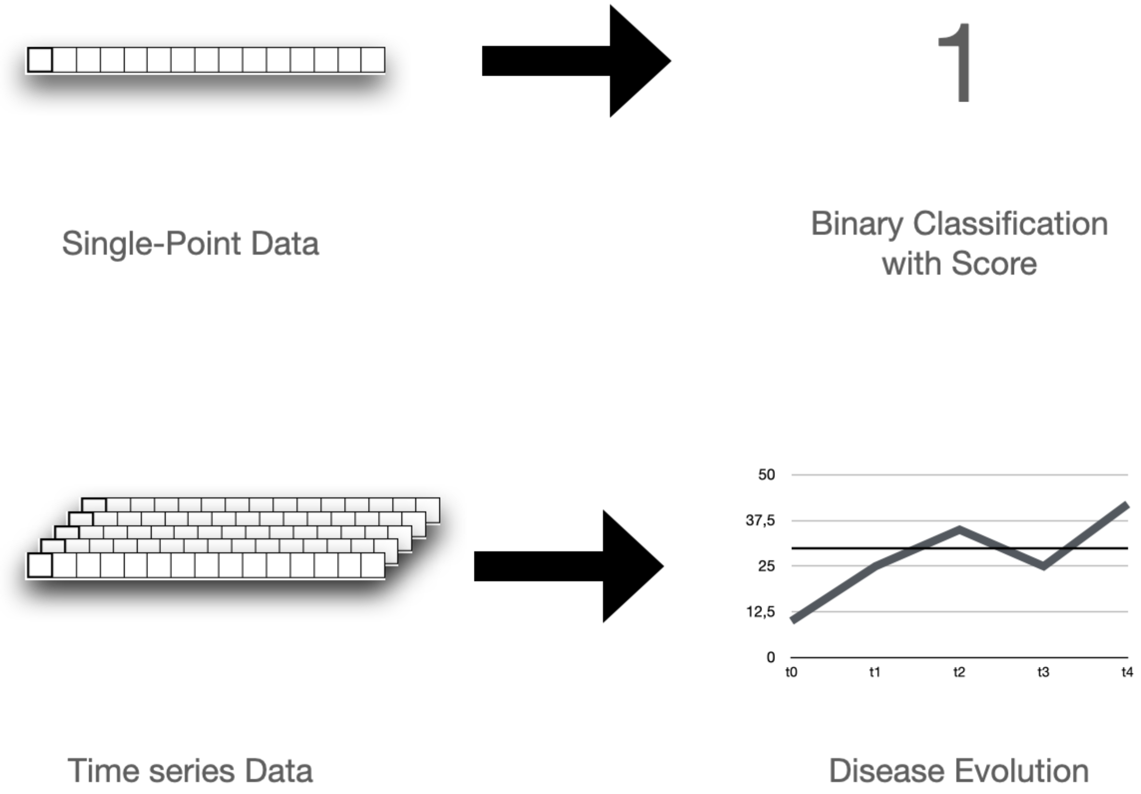

Fig S 8: Difference of paradigm between single-point analysis and longitudinal analysis

3.3 First Screening Tables

Number of links	1	2	3	4	5	6	7	8	9	10	11	12	13	14	15	16	17	18	19	20	21	22	23
Sequence classification Total	1029	210	83	42	28	13	6	6	6	6	NA	NA	NA	NA	NA	NA	NA	NA	NA	NA	NA	NA	NA
Sequence classification New	992	179	58	**21**	12	2	1	1	1	1	NA	NA	NA	NA	NA	NA	NA	NA	NA	NA	NA	NA	NA
Phenotype prediction Total	1069	239	116	72	63	59	54	50	49	48	47	46	42	42	42	39	34	31	30	30	21	NA	NA
Phenotype prediction New	1022	197	79	**37**	31	27	23	19	18	17	16	15	12	12	12	10	6	5	4	4	1	NA	NA
All papers Total	2065	512	215	129	98	85	67	61	59	58	52	51	49	47	47	46	38	38	34	34	28	28	23

All papers New	1979	433	147	**68**	46	34	26	21	19	18	17	16	15	13	13	12	6	6	4	4	1	1	0

Table S 7: Distribution of new articles discovered by number of original articles pointing to them. 0 means there were articles with such connectivity but none of them were new

Origin	Sequence Classification Only	Phenotype Prediction Only	All articles
Raw	21	37	68
Filtered	2	12	17

Table S 8: Distribution of new articles and kept new articles by origin

Taxonomy/Sequence Identification	Phenotype Classification	Miscellaneous
58	39	4

Table S 9: Distribution of articles by goal of the article.

raw reads	contigs	abundance table
36	10	48

Table S 10: Distribution of articles by type of input.

MLP	Autoencoders	CNN	RNN	Adversarial	NLP	Siamese	Graph embedding	ML Methods
28	17	31	8	3	20	3	2	20

Table S 11: Distribution of articles by Deep Learning methods.

Abundance	Mapping	sequence encoding	Image encoding	k-mer embedding	k-mer distribution	data augmentation	taxonomy	denoising	hashing	Co-presence	Time series	Reference database
46	10	14	5	8	16	3	14	3	4	7	8	15

Table S 12: Distribution of articles by features used.

Number of links	0	1	2	3	4	5	6	7	8	9	10	11	12	13	14	15	16	17	18	19	20	21	22	23	24	25	26	27	28	29	30	31	32	33	34
Number of new papers	2443	2443	638	260	**130**	94	73	55	40	32	27	23	22	20	19	17	14	13	12	12	9	6	5	5	5	4	4	3	3	3	2	2	1	1	1
Total of papers	2550	2550	736	343	**201**	162	136	118	100	92	84	72	68	66	58	56	53	52	50	50	47	42	39	39	39	37	37	36	34	34	32	32	30	30	30

Table S 13: Distribution of new articles discovered by number of original articles pointing to them in second screening. 0 means there were articles with such connectivity but none of them were new

Title	DOI	Publicly available	Date
Phylogenetic convolutional neural networks in metagenomics	10.1186/s12859-018-2033-5	Yes	March 8, 2018
Using convolutional neural networks to explore the microbiome	10.1109/EMBC.2017.8037799	Yes	July, 2017
Improved metagenome binning and assembly using deep variational autoencoders	10.1038/s41587-020-00777-4	Yes	January 4, 2021
Identifying viruses from metagenomic data using deep learning	10.1007/s40484-019-0187-4	Yes	March 8, 2020
CNN-MGP: Convolutional Neural Networks for Metagenomics Gene Prediction	10.1007/s12539-018-0313-4	Yes	December 11, 2021
DeepMicrobes: taxonomic classification for metagenomics with deep learning	10.1093/nargab/lqaa009	Yes	February 19, 2020
DeepARG: a deep learning approach for predicting antibiotic resistance genes from metagenomic data	10.1186/s40168-018-0401-z	Yes	February 1, 2021
PopPhy-CNN: A Phylogenetic Tree Embedded Architecture for Convolutional Neural Networks to Predict Host Phenotype From Metagenomic Data	10.1109/JBHI.2020.2993761	Yes	January 31, 2018
Deep learning models for bacteria taxonomic classification of metagenomic data	10.1186/s12859-018-2182-6	Yes	July 9, 2018
A deep siamese neural network improves metagenome-assembled genomes in microbiome datasets across different environments	10.1038/s41467-022-29843-y	Yes	April 28, 2022
Interpretable and accurate prediction models for metagenomics data	10.1093/gigascience/giaa010	Yes	March 1, 2021
Identification of antimicrobial peptides from the human gut microbiome using deep learning	10.1038/s41587-022-01226-0	No	March 3, 2022
MetaPheno: A critical evaluation of deep learning and machine learning in metagenome-based disease prediction	10.1016/j.ymeth.2019.03.003	Yes	August 15, 2019
Interpretable Machine Learning Framework Reveals Robust Gut Microbiome Features Associated With Type 2 Diabetes	10.2337/dc20-1536	Yes	December 7, 2020
Enhanced metagenomic deep learning for disease prediction and consistent signature recognition by restructured microbiome 2D representations	10.1016/j.patter.2022.100658	Yes	January 13, 2023
Human Gut Microbiome Aging Clock Based on Taxonomic Profiling and Deep Learning	10.1016/j.isci.2020.101199	Yes	May 23, 2020
phyLoSTM: a novel deep learning model on disease prediction from longitudinal microbiome data	10.1093/bioinformatics/btab482	Yes	July 2, 2021
Multimodal deep learning applied to classify healthy and disease states of human microbiome	10.1038/s41598-022-04773-3	Yes	January 17, 2022
A novel deep learning method for predictive modeling of microbiome data	10.1093/bib/bbaa073	Yes	May 20, 2021
Deep Learning for Metagenomic Data: using 2D Embeddings and Convolutional Neural Networks	10.48550/arXiv.1712.00244	Yes	December 1, 2017
Towards a metagenomics machine learning interpretable model for understanding the transition from adenoma to colorectal cancer	10.1038/s41598-021-04182-y	Yes	January 10, 2022
Machine learning and deep learning applications in microbiome research	10.1038/s43705-022-00182-9	Yes	October 6, 2022
Interpretable machine learning framework reveals microbiome features of oral disease	10.1101/2020.04.05.024984	Yes	December, 2022
Predicting microbiome compositions from species assemblages through deep learning	10.1002/imt2.3	Yes	March 1, 2022
CHEER: HierarCHical taxonomic classification for viral mEtagEnomic data via deep leaRning	10.1016/j.ymeth.2020.05.018	Yes	May 23, 2020
PPR-Meta: a tool for identifying phages and plasmids from metagenomic fragments using deep learning	10.1093/gigascience/giz066	Yes	June 20, 2019
MarkerML – Marker Feature Identification in Metagenomic Datasets Using Interpretable Machine Learning	10.1016/j.jmb.2022.167589	No	June 15, 2022
Taxonomic classification of metagenomic sequences from Relative Abundance Index profiles using deep learning	10.1016/j.bspc.2021.102539	No	May, 2021
CoCoNet: an efficient deep learning tool for viral metagenome binning	10.1093/bioinformatics/btab213	Yes	April 5, 2021
Disease Classification in Metagenomics with 2D Embeddings and Deep Learning	10.48550/arXiv.1806.09046	Yes	June 23, 2018
MetaVelvet-DL: a MetaVelvet deep learning extension for de novo metagenome assembly	10.1186/s12859-020-03737-6	Yes	June 2, 2021
Deep in the Bowel: Highly Interpretable Neural Encoder-Decoder Networks Predict Gut Metabolites from Gut Microbiome	10.1186/s12864-020-6652-7	Yes	July 20, 2020
Machine Learning and Deep Learning Applications in Metagenomic Taxonomy and Functional Annotation	10.3389/fmicb.2022.811495	Yes	March 14, 2022
Human microbiome aging clocks based on deep learning and tandem of permutation feature importance and accumulated local effects	10.1101/507780	Yes	December 28, 2018
Disease Prediction Using Synthetic Image Representations of Metagenomic Data and Convolutional Neural Networks	10.1109/RIVF.2019.8713670	No	May 16, 2019
PopPhy-CNN: A Phylogenetic Tree Embedded Architecture for Convolution Neural Networks for Metagenomic Data	10.1109/JBHI.2020.2993761	Yes	May 11, 2020
DeepToA: an ensemble deep-learning approach to predicting the theater of activity of a microbiome	10.1093/bioinformatics/btac584	Yes	August 27, 2022
Gene Prediction in Metagenomic Fragments with Deep Learning	10.1155/2017/4740354	Yes	November 8, 2017
Feature Extension of Gut Microbiome Data for Deep Neural Network-Based Colorectal Cancer Classification	10.1109/ACCESS.2021.3050838	Yes	January 11, 2021
A novel thermophilic chitinase directly mined from the marine metagenome using the deep learning tool Preoptem	10.1186/s40643-022-00543-1	Yes	May 16, 2022
Human disease prediction from microbiome data by multiple feature fusion and deep learning	10.1016/j.isci.2022.104081	Yes	April 15, 2022
MDITRE: Scalable and Interpretable Machine Learning for Predicting Host Status from Temporal Microbiome Dynamics	10.1128/msystems.00132-22	Yes	September 7, 2022
Novel taxonomy-independent deep learning microbiome approach allows for accurate classification of different forensically relevant human epithelial materials	10.1016/j.fsigen.2019.03.015	Yes	July, 2019
Application of Deep Learning in Microbiome	10.2991/jaims.d.201028.001	Yes	January, 2020
EnsDeepDP: An Ensemble Deep Learning Approach for Disease Prediction Through Metagenomics	10.1109/TCBB.2022.3201295	No	August 24, 2022
IDMIL: an alignment-free Interpretable Deep Multiple Instance Learning (MIL) for predicting disease from whole-metagenomic data	10.1093/bioinformatics/btaa477	Yes	July 13, 2020
Deep Learning Tools for Human Microbiome Big Data	10.1007/978-3-319-62521-8 ₋21	No	August, 2018
Utilizing longitudinal microbiome taxonomic profiles to predict food allergy via long short-term memory networks	10.1371/journal.pcbi.1006693	Yes	February 4, 2019
DLmeta: a deep learning method for metagenomic identification	10.1109/BIBM55620.2022.9995231	No	December 2022
Dimensionality Reduction for Cluster Identification in Metagenomics using Autoencoders	10.1109/ICTer51097.2020.9325447	Yes	November 2020
Stacking and Chaining of Normalization Methods in Deep Learning-Based Classification of Colorectal Cancer Using Gut Microbiome Data	10.1109/ACCESS.2021.3094529	Yes	July 2021
Metagenome-Based Disease Classification with Deep Learning and Visualizations Based on Self-organizing Maps	10.1007/978-3-030-35653-8 ₋20	No	November 2019
Ontology-aware neural network: a general framework for pattern mining from microbiome data	10.1093/bib/bbac005	Yes	March, 2022
Predicting Host Phenotype Based on Gut Microbiome Using a Convolutional Neural Network Approach	10.1007/978-1-0716-0826-5 ₋12	No	August 18, 2020
TopoPhy-CNN: Integrating Topological Information of Phylogenetic Tree for Host Phenotype Prediction From Metagenomic Data	10.1109/BIBM52615.2021.9669509	No	2021
NLP-MeTaxa: A Natural Language Processing Approach for Metagenomic Taxonomic Binning Based on Deep Learning	10.2174/1574893616666210621101150	Yes	June, 2021
Prediction of microbial communities for urban metagenomics using neural network approach	10.1186/s40246-019-0224-4	Yes	October 22, 2019
GraphKKE: graph Kernel Koopman embedding for human microbiome analysis	10.1007/s41109-020-00339-2	Yes	December 1, 2020
Understanding microbiome dynamics via interpretable graph representation learning	10.1038/s41598-023-29098-7	Yes	February 4, 2023
MegaD: Deep Learning for Rapid and Accurate Disease Status Prediction of Metagenomic Samples	10.3390/life12050669	Yes	April 30, 2022
G2S: A New Deep Learning Tool for Predicting Stool Microbiome Structure From Oral Microbiome Data	10.3389/fgene.2021.644516	Yes	April 9, 2021
Classification of Microbiome Data from Type 2 Diabetes Mellitus Individuals with Deep Learning Image Recognition	10.3390/bdcc7010051	Yes	March 17, 2023
Disease Prediction Using Metagenomic Data Visualizations Based on Manifold Learning and Convolutional Neural Network	10.1007/978-3-030-35653-8 ₋9	No	November 20, 2019
A self-knowledge distillation-driven CNN-LSTM model for predicting disease outcomes using longitudinal microbiome data	10.1093/bioadv/vbad059	Yes	May 18, 2023
PlasGUN: gene prediction in plasmid metagenomic short reads using deep learning	10.1093/bioinformatics/btaa103	Yes	May, 2020
Deep Learning Enables Design of Multifunctional Synthetic Human Gut Microbiome Dynamics	10.1101/2021.09.27.461983	Yes	September 28, 2021
ResMiCo: Increasing the quality of metagenome-assembled genomes with deep learning	10.1371/journal.pcbi.1011001	Yes	May 1, 2023
TAMPA: interpretable analysis and visualization of metagenomics-based taxon abundance profiles	10.1093/gigascience/giad008	Yes	February 28, 2023
Deep Learning Encoding for Rapid Sequence Identification on Microbiome Data	10.3389/fbinf.2022.871256	Yes	June 24, 2022
Using Autoencoders for Predicting Latent Microbiome Community Shifts Responding to Dietary Changes	10.1109/BIBM47256.2019.8983124	Yes	November, 2019
Early Identification of Fungal and Mycobacterium Infections in Pulmonary Granulomas Using Metagenomic Next-Generation Sequencing on Formalin fixation and paraffin embedding tissue	10.1080/14737159.2022.2052046	No	April 22, 2022
Life Language Processing: Deep Learning-based Language-agnostic Processing of Proteomics, Genomics/Metagenomics, and Human Languages	/	Yes	2019
SemiBin: Incorporating information from reference genomes with semi-supervised deep learning leads to better metagenomic assembled genomes (MAGs)	10.1101/2021.08.16.456517	Yes	August 16, 2021
Deep learning and host variable embedding augment microbiome-based simultaneous detection of multiple diseases	10.1101/2023.05.16.541058	Yes	May 18, 2023
NLP-based classification of software tools for metagenomics sequencing data analysis into EDAM semantic annotation	10.48550/arXiv.2210.00831	Yes	September 30, 2022
Reject and Cascade Classifier with Subgroup Discovery for Interpretable Metagenomic Signatures	10.1007/978-3-030-93736₋2 5	Yes	February 17, 2022
MT-MAG: Accurate and interpretable machine learning for complete or partial taxonomic assignments of metagenome-assembled genomes	10.1101/2022.01.12.475159	Yes	June 21, 2023
GMEmbeddings: An R Package to Apply Embedding Techniques to Microbiome Data	10.3389/fbinf.2022.828703	Yes	April 26, 2022
Improving Disease Prediction using Shallow Convolutional Neural Networks on Metagenomic Data Visualizations based on Mean-Shift Clustering Algorithm	10.14569/IJACSA.2020.0110607	Yes	January, 2020
DeepMicro: deep representation learning for disease prediction based on microbiome data	10.1038/s41598-020-63159-5	Yes	April 7, 2020
MetaMLP: A Fast Word Embedding Based Classifier to Profile Target Gene Databases in Metagenomic Samples	10.1089/cmb.2021.0273	No	November 28, 2021
A Deep Learning Approach to Predict Health Status Using Microbiome Profiling	10.1109/ICTer58063.2022.10024102	No	November 30, 2022
Adversarial and variational autoencoders improve metagenomic binning	10.1101/2023.02.27.527078	Yes	February 27, 2023
Interpretable machine learning analysis of functional metagenomic profiles improves colorectal cancer prediction and reveals basic molecular mechanisms	10.21203/rs.2.21634/v1	Yes	January, 2020
Neural network-based taxonomic clustering for metagenomics	10.1109/IJCNN.2010.5596644	Yes	July, 2010
Embedding the de Bruijn graph, and applications to metagenomics	10.1101/2020.03.06.980979	Yes	March 8, 2020
Interpretable Machine Learning Algorithm Reveals Novel Gut Microbiome Features in Predicting Type 2 Diabetes	10.1093/cdn/nzaa062₋016	Yes	June, 2020
Metagenomic Binning using Connectivity-constrained Variational Autoencoders	id8OSRGeew	Yes	April 28, 2023
Deep Learning Approach for Pathogen Detection Through Shotgun Metagenomics Sequence Classification	10.1007/978-3-030-21642-9 ₋4	No	May 30, 2019
Feature Selection Based on a Shallow Convolutional Neural Network and Saliency Maps on Metagenomic Dat	10.1007/978-981-33-6385-4 ₋10	No	January, 2021
Metagenomic Sequence Classification based on One-Dimensional Convolutional Neural Network	10.1145/3581807.3581835	Yes	2022
A K-mer based Multi Convolutional Neural Network Classifier of Low-Ranking Taxonomic Bins from Metagenome	335540811	Yes	September, 2019
Feature Selection Using Local Interpretable Model-Agnostic Explanations on Metagenomic Data	10.1007/978-3-030-76620-7 ₋6	No	June, 2021
DeephageTP: a convolutional neural network framework for identifying phage-specific proteins from metagenomic sequencing data	10.7717/peerj.13404	Yes	June 8, 2022
A neural network-based framework to understand the type 2 diabetes-related alteration of the human gut microbiome	10.1002/imt2.20	Yes	May 5, 2022
From mechanism to application: decrypting light-regulated denitrifying microbiome through geometric deep learning	10.21203/rs.3.rs-3086818/v1	No	June, 2023
MT-MAG: Accurate and interpretable machine learning based taxonomic assignment of metagenome-assembled genomes, with a partial classification option	10.1101/2022.01.12.475159	Yes	June 21, 2023
A Virtual Machine Platform for Non-Computer Professionals for Using Deep Learning to Classify Biological Sequences of Metagenomic Data	10.3791/62250	No	September 25, 2021
Deep Learning for Metagenomic Data: using 2D Embeddings and Convolutional Neural Networks	10.48550/arXiv.1712.00244	Yes	December, 2017
Ontology-aware deep learning for antibiotic resistance gene prediction: novel function discovery and comprehensive profiling from metagenomic data	10.1093/bib/bbac005	Yes	March, 2022
A Novel Metagenomic Binning Framework Using NLP Techniques in Feature Extraction	10.2197/ipsjtbio.15.1	Yes	January, 2022
MEGGASENSE – The Metagenome/Genome Annotated Sequence Natural Language Search Engine: A Platform for the Construction of Sequence Data Warehouses	10.17113/ftb.55.02.17.4749	Yes	June, 2017
Machine learning and deep learning applications in microbiome research	10.1038/s43705-022-00182-9	Yes	October 6, 2022
Scalable learning of interpretable rules for the dynamic microbiome domain	10.1101/2020.06.25.172270	Yes	June 28, 2022
Enhancing Microbiome Host Disease Prediction with Variational Autoencoders	10.36837/chapman.000297	Yes	July 1, 2021
Marginalised stack denoising autoencoders for metagenomic data binning	10.1109/CIBCB.2017.8058552	Yes	August, 2017
Human Gut Microbiome Data Analysis for Disease Likelihood Prediction Using Autoencoders	10.1109/ICter53630.2021.9774811	No	December, 2021
MT-MAG: Accurate and interpretable machine learning for complete or partial taxonomic assignments of metagenome-assembled genomes	10.1101/2022.01.12.475159	Yes	January 12, 2022
Deep Neural Network Modeling for Phenotypic Prediction of Metagenomic Samples	10.1145/3388440.3414921	Yes	November 10, 2022
A Fast Word Embedding Based Classifier to Profile Target Gene Databases in Metagenomic Samples	10.1007/978-3-030-79290-9 ₋10	No	July 3, 2021
Reference-free biomarker mining in metagenomic data using language embedding	ad1c6a14-d8a0-4973-9eae-fac46b0bdf9c	Yes	April 21, 2021
Brain neural network, development, microbiome, microbial toxins and COVID-19	10.52547/jcbior.3.1.14	Yes	2022
Efficient and Interpretable Machine Learning Algorithms for Predictive Analyses in Metagenomic Data	10.21203/rs.2.21634/v1	Yes	January, 2020
Scalable learning of interpretable rules for the dynamic microbiome domain	10.1101/2020.06.25.172270	Yes	June 28, 2020
Enhanced Metagenomic Deep Learning for Disease Prediction and Reproducible Signature Identification by Restructured Microbiome 2D-Representations	10.1016/j.patter.2022.100658	No	January 13, 2023
Taxonomic Profiling of Metagenomic Reads Using Deep Learning	10.23860/thesis-Cres-Cecile-2020	Yes	2020
A novel deep learning pipeline for early detection of colorectal cancer and colorectal adenoma using gut microbiome data	10.1158/1538-7445.AM2023-3032	No	April 4, 2023
Deep learning for microbiome-based disease prediction and rheumatoid arthritis hand joint detection	1993/37128	No	December 15, 2022
Efficient Sequence Clustering and Embedding Algorithms for Large-scale Metagenomics Data	13884437	Yes	2019
Some contributions to deep learning for metagenomcs	tel-01958389v1	Yes	September 26, 2018
Representation counts: the impact of embedding models on disease detection tasks from microbiome sequencing data	c0aaa51a-18b3-4e2a-b507-9cb4bd7c08a9	Yes	June 23, 2022
NLP-based classification of software tools for metagenomics sequencing data analysis into EDAM semantic annotation	10.48550/arXiv.2210.00831	Yes	October 18, 2022
Multimodal deep learning models to classify healthy and disease states of human microbiome	10.1038/s41598-022-04773-3	Yes	January 17, 2022
Advances in Microbiome Analysis: From the Variance Component Model to Deep Learning	13886596	Yes	2019
Deep learning-based colorectal cancer classification using augmented and normalised gut microbiome data	14415	Yes	2022
DeepBiome: a phylogenetic tree informed deep neural network for microbiome data analysis	10.21203/rs.3.rs-33860/v1	Yes	June 18, 2020
Deep learning approach to metagenomic binning	1721.1/119755	Yes	2018
Deep Learning Frameworks for Multi-Omics Analyses of the Microbiome in Disease Studies	19187285	Yes	December 1, 2021
Predicting the Complexity and Progression of the Gut Microbiome Using Temporal Data and Deep Learning	8pt8g0bm	Yes	2019
Novel Mediation Method for Metagenomic Studies and Deep Learning Methods for Single Cell RNA-Seq Studies	28258769	Yes	2020
LA2SNE: A novel stochastic neighbor embedding approach for microbiome data visualization	10.1109/BIBM.2014.6999294	No	2014
A virtual machine platform for non-computer professionals for using deep learning to classify biological sequences of metagenomic data	10.3791/62250	No	September 25, 2021
Disease Classification in Metagenomics with 2D Embeddings and Deep Learning	10.48550/arXiv.1806.09046	Yes	June 23, 2018
Inferring Gut Microbial Interaction Network from Microbiome Data Using Network Embedding Algorithm	10.12146/j.issn.2095-3135.20190704001	Yes	October 9, 2019
Constructing Long Short-Term Memory Networks to Predict Ulcerative Colitis Progression from Longitudinal Gut Microbiome Profiles	10.33137/utjph.v2i2.36763	Yes	September 5, 2021
Female Urinary Metagenomic Analysis and Natural Language Processing Enhances the Infectious Diagnostic Yield in Precision Medicine	10.1093/ofid/ofaa439.1859	Yes	October 7, 2020
META2: Memory-efficient taxonomic classification and abundance estimation for metagenomics with deep learning	10.48550/arXiv.1909.13146	Yes	February 10, 2020
Deep Multiple Instance Learning for Taxonomic classification of metagenomic read sets	10.48550/arXiv.1909.13146	Yes	February 10, 2021
Some contributions to deep learning for metagenomics	tel-01958389v1	Yes	September 26, 2018
Accurate identification of bacteriophages from metagenomic data using Transformer	10.1093/bib/bbac258	Yes	June 30, 2022
ViBE: a hierarchical BERT model to identify eukaryotic viruses using metagenome sequencing data	10.1093/bib/bbac204	No	2022
A Deep Embedded Clustering Algorithm for the Binning of Metagenomic Sequences	10.1109/ACCESS.2022.3176954	Yes	May 23, 2022
Virsearcher: Identifying Bacteriophages from Metagenomes by Combining Convolutional Neural Network and Gene Information	10.1109/TCBB.2022.3161135	No	2023
Phylogeny-Aware Deep 1-Dimensional Convolutional Neural Network for the Classification of Metagenomes	10.1109/BIBM.2018.8621543	No	January 24, 2019
RNN-VirSeeker: A Deep Learning Method for Identification of Short Viral Sequences From Metagenomes	10.1109/TCBB.2020.3044575	Yes	June, 2022
CLMB: deep contrastive learning for robust metagenomic binning	10.48550/arXiv.2111.09656	Yes	November 18, 2021
Multi-Layer and Recursive Neural Networks for Metagenomic Classification	10.1109/TNB.2015.2461219	Yes	September 6, 2015
Graph Embedding Deep Learning Guides Microbial Biomarkers' Identification	10.3389/fgene.2019.01182	Yes	November 22, 2019
Enhancing Metagenome-based Disease Prediction by Unsupervised Binning Approaches	10.1109/KSE.2019.8919295	No	October, 2019
MicroPheno: predicting environments and host phenotypes from 16S rRNA gene sequencing using a k-mer based representation of shallow sub-samples	10.1093/bioinformatics/bty296	Yes	June 27, 2018
Diagnosis Approaches for Colorectal Cancer Using Manifold Learning and Deep Learning	10.1007/s42979-020-00297-7	No	August 27, 2020
TaxoNN: ensemble of neural networks on stratified microbiome data for disease prediction	10.1093/bioinformatics/btaa542	Yes	May 25, 2020
Multiclass Disease Classification from Microbial Whole-Community Metagenomes using Graph Convolutional Neural Networks	10.1101/726901	Yes	August 16, 2019
Model-free prediction of microbiome compositions	10.1101/2022.02.04.479107	Yes	February 4, 2022
DETIRE: a hybrid deep learning model for identifying viral sequences from metagenomes	10.3389/fmicb.2023.1169791	Yes	June 16, 2023
A Framework for Effective Application of Machine Learning to Microbiome-Based Classification Problems	10.1128/mbio.00434-20	Yes	June 9, 2022
Taxonomy-aware feature engineering for microbiome classification	10.1186/s12859-018-2205-3	Yes	June 15, 2018
Resource saving taxonomy classification with k-mer distributions and machine learning	10.48550/arXiv.2303.06154	Yes	March 10, 2023
Enhancing Disease Prediction on Imbalanced Metagenomic Dataset by Cost-Sensitive	10.14569/IJACSA.2020.0110778	Yes	2020
Applications of Machine Learning in Human Microbiome Studies: A Review on Feature Selection, Biomarker Identification, Disease Prediction and Treatment	10.3389/fmicb.2021.634511	Yes	February 19, 2021
MITRE: predicting host status from microbiota time-series data	10.1186/s13059-019-1788-y	Yes	September 2, 2019
Efficient Discretization Approaches For Machine Learning Techniques To Improve Disease Classification On Gut Microbiome Composition Data	10.25046/aj050368	Yes	January, 2020
The influence of machine learning technologies in gut microbiome research and cancer studies - A review	10.1016/j.lfs.2022.121118	Yes	December 15, 2022
VirNet: Deep attention model for viral reads identification	10.1109/ICCES.2018.8639400	No	December, 2018
The Phylogenetic Tree based Deep Forest for Metagenomic Data Classification	10.1109/BIBM.2018.8621463	No	December, 2018
An Ensemble Feature Selection Method Based on Deep Forest for Microbiome-Wide Association Studies	10.1109/BIBM.2018.8621461	No	January, 2019
Gene Family Abundance Visualization based on Feature Selection Combined Deep Learning to Improve Disease Diagnosis	10.5614/j.eng.technol.sci.2021.53.1.9	No	January 30, 2021
Effective Disease Prediction on Gene Family Abundance Using Feature Selection and Binning Approach	10.1007/978-981-15-9354-3 2	No	December 8, 2020

Table S 14: **Table of data availability.** This table contains the studies synthesized in this work, along with their date of publication, their id and whether they are publicly available or not.

3.4. Connectivity of newly found papers

This paragraph concerns the first screening, happening in July 2022. The new relevant articles we selected with the Connected Papers approach had a co- citation connectivity greater than 4. However, we can question whether there is a difference between retained and filtered articles in terms of co-citation connectivity. Therefore, we will know if articles who passed the abstract filtering were more connected than the ones who did not pass this check. We plotted the proportion for each co-citation connectivity in our three datasets, the result can be seen in Figure S4. Although the number of retained articles is quite low, it seems that co-citation connectivity did not influence the relevance of an article. The distributions have close means (8 and 8.41 for ”All” and ”All After Filtering (AF)”, 10.68 and 10 for ”Phenotype Prediction” and ”Phenotype Prediction AF”,
and finally 5.05 and 4 for ”Sequence Classification” and ”Sequence Classification AF”). We may note that most of the cited articles did not pass the filtering. This could be due to the fact that many cited articles tend to be more general and therefore may not be related to both domains. There is an environment of articles that are found many times but actually do not link both our topics

References

[1] Bao HQ, Vinh LV, Van Hoai T. A Deep Embedded Clustering Algorithm for the Binning of Metagenomic Sequences. IEEE Access. 2022;10:54348–54357. doi:10.1109/ACCESS.2022.3176954.

[2] Busia A, Dahl GE, Fannjiang C, Alexander DH, Dorfman E, Poplin R, et al. A deep learning approach to pattern recognition for short DNA sequences; p. 12.

[3] Matougui B, Batouche M, Boukelia A. A K-mer based Multi Convolutional Neural Network Classifier of Low-Ranking Taxonomic Bins from Metagenome; p. 13.

[4] Wijegunarathna K, Maduranga U, Weerasinghe S, Perera I, Wickaramarachchi A. Cluster Identification in Metagenomis – A Novel Tech-

[5] Maduranga U, Wijegunarathna K, Weerasinghe S, Perera I, Wickramarachchi A. Dimensionality Reduction for Cluster Identification in Metagenomics using Autoencoders. In: 2020 20th International Conference on Advances in ICT for Emerging Regions (ICTer). Colombo, Sri Lanka: IEEE; 2020. p. 113–118. Available from: https://ieeexplore.ieee.org/document/9325447/.

[6] Menegaux R, Vert JP. Embedding the de Bruijn graph, and applications to metagenomics. Bioinformatics; 2020. Available from: http://biorxiv.org/lookup/doi/10.1101/2020.03.06.980979.

[7] Rojas-Carulla M, Tolstikhin I, Luque G, Youngblut N, Ley R, Scho¨lkopf B. GeNet: Deep Representations for Metagenomics; p. 13.

[8] Kouchaki S, Tirunagari S, Tapinos A, Robertson DL. Marginalised stack denoising autoencoders for metagenomic data binning. In: 2017 IEEE Conference on Computational Intelligence in Bioinformatics and Computational Biology (CIBCB). Manchester, United Kingdom: IEEE; 2017. p. 1–6. Available from: http://ieeexplore.ieee.org/document/8058552/.

[9] Georgiou A, Fortuin V, Mustafa H, Rätsch G. META$ˆ\mathbf{2}$: Memory-efficient taxonomic classification and abundance estimation for metagenomics with deep learning; 2020. Available from: http://arxiv.org/abs/1909.13146.

[10] Liang Kc. MetaVelvet-DL: a MetaVelvet deep learning extension for de novo metagenome assembly. 2021; p. 21.

[11] Matougui B, Boukelia A, Belhadef H, Galiez C, Batouche M. NLP-MeTaxa: A Natural Language Processing Approach for Metagenomic Taxonomic

Binning Based on Deep Learning. Current Bioinformatics. 2021;16(7):992– 1003. doi:10.2174/1574893616666210621101150.

[12] Mock F, Kretschmer F, Kriese A, Bo¨cker S, Marz M. BERTax: taxonomic classification of DNA sequences with Deep Neural Networks. Bioinformatics; 2021. Available from: http://biorxiv.org/lookup/doi/10.1101/2021.07.09.451778.

[13] Karagöz MA, Nalbantoglu OU. Taxonomic classification of metage- nomic sequences from Relative Abundance Index profiles using deep learning. Biomedical Signal Processing and Control. 2021;67:102539. doi:10.1016/j.bspc.2021.102539.

[14] Nissen JN, Johansen J, Allesøe RL, Sønderby CK, Armenteros JJA, Grønbech CH, et al. Improved metagenome binning and assembly using deep variational autoencoders. Nature Biotechnology. 2021;39(5):555–560. doi:10.1038/s41587-020-00777-4.

[15] Zhang P, Jiang Z, Wang Y, Li Y. CLMB: deep contrastive learning for robust metagenomic binning; p. 20.

[16] Pan S, Zhu C, Zhao XM, Coelho LP. A deep siamese neural network improves metagenome-assembled genomes in microbiome datasets across different en- vironments. Nature Communications. 2022;13(1):2326. doi:10.1038/s41467-022-29843-y.

[17] Kang DD, Li F, Kirton E, Thomas A, Egan R, An H, et al. MetaBAT 2: an adaptive binning algorithm for robust and efficient genome reconstruction from metagenome assemblies. PeerJ. 2019;7:e7359. doi:10.7717/peerj.7359.

[18] Piera Lindez P, Johansen J, Sigurdsson AI, Nissen JN, Rasmussen S. Ad- versarial and variational autoencoders improve metagenomic binning. Bioin-

formatics; 2023. Available from: http://biorxiv.org/lookup/doi/10.1101/2023.02.27.527078.

[19] Lamurias A, Tibo A, Hose K, Albertsen M, Nielsen TD. Metagenomic Binning using Connectivity-constrained Variational Autoencoders;.

[20] Guo S, Zhang H, Chu Y, Jiang Q, Ma Y. A neural network-based framework to understand the type 2 diabetes-related alteration of the human gut microbiome. iMeta. 2022;1(2). doi:10.1002/imt2.20.

[21] Phan NYK, Nguyen HT. Binning on Metagenomic Data for Disease Prediction Using Linear Discriminant Analysis and K-Means. In: Anh NL, Koh SJ, Nguyen TDL, Lloret J, Nguyen TT, editors. Intelligent Systems and Networks. vol. 471. Singapore: Springer Nature Singapore; 2022. p. 402–409. Available from: https://link.springer.com/10.1007/978-981-19-3394-3_46.

[22] Tataru C, Eaton A, David MM. GMEmbeddings: An R Package to Apply Embedding Techniques to Microbiome Data. Frontiers in Bioinformatics. 2022;2:828703. doi:10.3389/fbinf.2022.828703.

[23] Shen Y, Zhu J, Deng Z, Lu W, Wang H. Ensdeepdp: An Ensemble Deep Learning Approach for Disease Prediction Through Metagenomics. IEEE/ACM Transactions on Computational Biology and Bioinformatics. 2022; p. 1–14. doi:10.1109/TCBB.2022.3201295.

[24] Oh M, Zhang L. DeepMicro: deep representation learning for disease prediction based on microbiome data. Scientific Reports. 2020;10(1):6026. doi:10.1038/s41598-020-63159-5.

[25] Lo C, Marculescu R. MetaNN: accurate classification of host pheno- types from metagenomic data using neural networks. BMC Bioinformatics. 2019;20(S12):314. doi:10.1186/s12859-019-2833-2.

[26] Nguyen TH, Phan TT, Dao CT, Ta DVP, Nguyen TNC, Phan NMT, et al. Effective Disease Prediction on Gene Family Abundance Using Feature Selection and Binning Approach. In: Kim H, Kim KJ, editors. IT Convergence and Security. vol. 712. Singapore: Springer Singapore; 2021. p. 19–28. Available from: http://link.springer.com/10.1007/978-981-15-9354-3_2.

[27] Mulenga M, Kareem SA, Sabri AQ. Stacking and Chaining of Normalization Methods in Deep Learning-Based Classification of Colorectal Cancer Using Gut Microbiome Data. 2021;9:24.

[28] Rahman MA, Rangwala H. IDMIL: an alignment-free Interpretable Deep Multiple Instance Learning (MIL) for predicting disease from whole-metagenomic data; p. 9.

[29] Queyrel M, Prifti E, Templier A, Zucker JD. Towards end-to-end disease prediction from raw metagenomic data. Genomics; 2020. Available from: http://biorxiv.org/lookup/doi/10.1101/2020.10.29.360297.

[30] Strocchi M, Corso G, Lio` P. Representation counts: the impact of embedding models on disease detection tasks from microbiome sequencing data; p. 12.

[31] Asgari E, Garakani K, McHardy AC, Mofrad MRK. MicroPheno: predict- ing environments and host phenotypes from 16S rRNA gene sequencing using a k-mer based representation of shallow sub-samples. Bioinformatics. 2018;34(13):i32–i42. doi:10.1093/bioinformatics/bty296.

[32] Lee SJ, Rho M. Multimodal deep learning applied to classify healthy and disease states of human microbiome. Scientific Reports. 2022;12(1):824. doi:10.1038/s41598-022-04773-3.

[33] Reiman D, Metwally AA, Sun J, Dai Y. PopPhy-CNN: A Phylogenetic Tree Embedded Architecture for Convolutional Neural Networks to Predict Host Phenotype From Metagenomic Data. IEEE Journal of Biomedical and Health Informatics. 2020;24(10):2993–3001. doi:10.1109/JBHI.2020.2993761.

[34] Fioravanti D, Giarratano Y, Maggio V, Agostinelli C, Chierici M, Jurman G, et al. Phylogenetic convolutional neural networks in metagenomics. BMC Bioinformatics. 2018;19(S2):49. doi:10.1186/s12859-018-2033-5.

[35] Sharma D, Paterson AD, Xu W. TaxoNN: ensemble of neural networks on stratified microbiome data for disease prediction. Bioinformatics. 2020;36(17):4544–4550. doi:10.1093/bioinformatics/btaa542.

[36] Zhu Q, Jiang X, Zhu Q, Pan M, He T. Graph Embedding Deep Learning Guides Microbial Biomarkers' Identification. Frontiers in Genetics. 2019;10:1182. doi:10.3389/fgene.2019.01182.

[37] Chen X, Zhu Z, Zhang W, Wang Y, Wang F, Yang J, et al. Human disease prediction from microbiome data by multiple feature fusion and deep learning. iScience. 2022;25(4):104081. doi:10.1016/j.isci.2022.104081.

[38] Khan S, Kelly L. Multiclass Disease Classification from Microbial Whole-Community Metagenomes using Graph Convolutional Neural Networks. Bioinformatics; 2019. Available from: http://biorxiv.org/lookup/doi/10.1101/726901.

[39] Shen WX, Liang SR, Jiang YY, Chen YZ. Enhanced metagenomic deep learning for disease prediction and consistent signature recognition by

restructured microbiome 2D representations. Patterns. 2023;4(1):100658. doi:10.1016/j.patter.2022.100658.

[40] Nguyen TH, Prifti E, Chevaleyre Y, Sokolovska N, Zucker JD. Disease Classification in Metagenomics with 2D Embeddings and Deep Learning. arXiv:180609046 [cs]. 2018;.

[41] Nguyen HT, Bao T, Hoang H, Phuoc T, C N. Improving Disease Pre- diction using Shallow Convolutional Neural Networks on Metagenomic Data Visualizations based on Mean-Shift Clustering Algorithm. Interna- tional Journal of Advanced Computer Science and Applications. 2020;11(6). doi:10.14569/IJACSA.2020.0110607.

[42] Pfeil J, Siptroth J, Pospisil H, Frohme M, Hufert FT, Moskalenko O, et al. Classification of Microbiome Data from Type 2 Diabetes Mellitus Individuals with Deep Learning Image Recognition. Big Data and Cognitive Computing. 2023;7(1):51. doi:10.3390/bdcc7010051.

[43] Mreyoud Y, Song M, Lim J, Ahn TH. MegaD: Deep Learning for Rapid and Accurate Disease Status Prediction of Metagenomic Samples. Life. 2022;12(5):669. doi:10.3390/life12050669.

[44] Fung DLX, Li X, Leung CK, Hu P. A self-knowledge distillation- driven CNN-LSTM model for predicting disease outcomes using longi- tudinal microbiome data. Bioinformatics Advances. 2023;3(1):vbad059. doi:10.1093/bioadv/vbad059.
